# Metal Complexes as Antifungals? From a Crowd-Sourced Compound Library to the First *In Vivo* Experiments

**DOI:** 10.1021/jacsau.2c00308

**Published:** 2022-09-23

**Authors:** Angelo Frei, Alysha G. Elliott, Alex Kan, Hue Dinh, Stefan Bräse, Alice E. Bruce, Mitchell R. Bruce, Feng Chen, Dhirgam Humaidy, Nicole Jung, A. Paden King, Peter G. Lye, Hanna K. Maliszewska, Ahmed M. Mansour, Dimitris Matiadis, María Paz Muñoz, Tsung-Yu Pai, Shyam Pokhrel, Peter J. Sadler, Marina Sagnou, Michelle Taylor, Justin J. Wilson, Dean Woods, Johannes Zuegg, Wieland Meyer, Amy K. Cain, Matthew A. Cooper, Mark A. T. Blaskovich

**Affiliations:** † Centre for Superbug Solutions, Institute for Molecular Bioscience, The University of Queensland, St. Lucia, Queensland4072, Australia; ‡ Department of Chemistry, Biochemistry & Pharmaceutical Sciences, University of Bern, Freiestrasse 3, 3012Bern, Switzerland; § Molecular Mycology Research Laboratory, Centre for Infectious Diseases and Microbiology, Faculty of Medicine and Health, Sydney Medical School, Westmead Clinical School, Sydney Institute for Infectious Diseases, Westmead Hospital-Research and Education Network, Westmead Institute for Medical Research, University of Sydney, Sydney, NSW2145, Australia; ∥ School of Natural Sciences, ARC Centre of Excellence in Synthetic Biology, Macquarie University, Sydney, NSW2109, Australia; ⊥ Institute of Organic Chemistry, Karlsruhe Institute of Technology, Fritz-Haber-Weg 6, 76131Karlsruhe, Germany; # Karlsruhe Nano Micro Facility (KNMF), Karlsruhe Institute of Technology, Hermann-von-Helmholtz-Platz 1, 76344Eggenstein-Leopoldshafen, Germany; ∇ Department of Chemistry, University of Maine, Orono, Maine04469, United States; ○ Department of Chemistry, University of Warwick, Gibbet Hill Road, CoventryCV4 7AL, U.K.; ◆ Department of Chemistry and Chemical Biology, Cornell University, Ithaca, New York14853, United States; ¶ School of Science and Technology, University of New England, Armidale, NSW2351, Australia; & School of Chemistry, University of East Anglia, Norwich Research Park, NorwichNR4 7TJ, U.K.; ● Chemistry Department, Faculty of Science, Cairo University, Giza12613, Egypt; ◊ Institute of Biosciences & Applications, National Centre for Scientific Research “Demokritos”, 15310Athens, Greece; □ Institute of Biological and Chemical Systems - Functional Molecular Systems, Karlsruhe Institute of Technology, 76344Eggenstein-Leopoldshafen, Germany

**Keywords:** metal complexes, antifungal, antimicrobial resistance, inorganic, organometallic, antimycotic

## Abstract

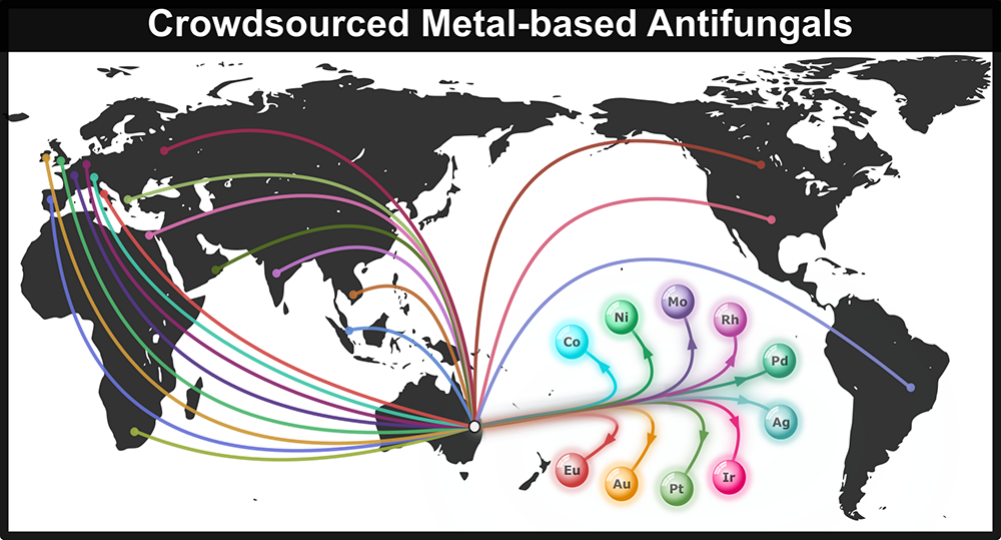

There are currently fewer than 10 antifungal drugs in clinical development, but new fungal strains that are resistant to most current antifungals are spreading rapidly across the world. To prevent a second resistance crisis, new classes of antifungal drugs are urgently needed. Metal complexes have proven to be promising candidates for novel antibiotics, but so far, few compounds have been explored for their potential application as antifungal agents. In this work, we report the evaluation of 1039 metal-containing compounds that were screened by the Community for Open Antimicrobial Drug Discovery (CO-ADD). We show that 20.9% of all metal compounds tested have antimicrobial activity against two representative *Candida* and *Cryptococcus* strains compared with only 1.1% of the >300,000 purely organic molecules tested through CO-ADD. We identified 90 metal compounds (8.7%) that show antifungal activity while not displaying any cytotoxicity against mammalian cell lines or hemolytic properties at similar concentrations. The structures of 21 metal complexes that display high antifungal activity (MIC ≤1.25 μM) are discussed and evaluated further against a broad panel of yeasts. Most of these have not been previously tested for antifungal activity. Eleven of these metal complexes were tested for toxicity in the *Galleria mellonella* moth larva model, revealing that only one compound showed signs of toxicity at the highest injected concentration. Lastly, we demonstrated that the organo-Pt(II) cyclooctadiene complex **Pt1** significantly reduces fungal load in an *in vivoG. mellonella* infection model. These findings showcase that the structural and chemical diversity of metal-based compounds can be an invaluable tool in the development of new drugs against infectious diseases.

## Introduction

Fungal infections are currently widely overlooked, failing to attract attention despite a recent focus on the antibacterial drug crisis. However, it is well-documented that fungal infections are proliferating around the world with an estimated 1.5 million deaths per year.^1−3^ While healthy humans are generally not affected by fungal infections, they are a major concern to immunocompromised individuals.^4^ Modern medical treatments such as chemotherapy, transplantations, and broad-spectrum antibiotic courses lead to an increased population of people susceptible to fungal infections.^5^ Of particular concern are *Candida, Aspergillus*, and *Cryptococcus* species, which are responsible for >90% of fungal infection deaths. *Candida* species, and *Candida albicans* in particular, are the most prevalent cause of healthcare-associated bloodstream infections in the US. Despite the availability of antifungal drugs, these infections have a mortality rate of around 40%.^3^ Over the last few years, *Candida glabrata* has caused a continuously increasing number of identified cases,^6^ while *Candida auris*, first reported in 2009, has already been identified in over 30 countries across 6 continents. *C. auris* generally displays resistance against at least one class of antifungal drugs and, in some cases, resistance against all three major antifungal drug classes (polyenes, azoles, and echinocandins),^7^ leading to the 2019 Centers for Disease Control and Prevention (CDC) report on ″Antibiotic Resistance Threats in the United States″ listing *C. auris* as an ″Urgent Threat″. Reported cases increased 318% in 2018 when compared to the average number of cases reported in 2015 to 2017.^8^

Despite the growing threat of fungal infections to global health, the current development pipeline for antifungal agents is even sparser than the already meager antibacterial drug landscape, with fewer than 10 drugs in various phases of clinical development.^5,9^ Promisingly, some of these compounds represent new classes with novel modes of action. However, with the high attrition rates of compounds during clinical trials, few can be expected to be approved for clinical use in the coming years. To prevent drug resistance from overwhelming the capabilities of the global healthcare system, a richer and more diverse antifungal drug pipeline is urgently needed.

While all current antifungal drugs and antifungal drug candidates are exclusively organic molecules, metal-containing compounds have been a cornerstone of medicine since the beginning of the 20th century. Today, metal complexes are present mainly in the field of anticancer therapies where platinum-based drugs (e.g., cisplatin) are still the most frequently used chemotherapeutics despite having been introduced over 40 years ago.^10^ Since then, metal compounds that contain titanium, iron, copper, gallium, molybdenum, ruthenium, palladium, silver, gold, and bismuth have entered clinical trials.^11^ Indeed, in 2020, 12 metal complexes were in clinical trials for anticancer indications alone.^12^ In the field of infectious diseases, the iron-based antimalaria drug-candidate ferroquine advanced to phase II clinical trials, though it was not successful.^13^ Recently, a spin-off company was established to advance a series of dinuclear ruthenium complexes with promising antimicrobial properties.^14−18^

While metal-based drugs are still a niche field, interest in their clinical use is increasing. Metal complexes offer two potentially advantageous properties that set them apart from their purely organic counterparts. First, the various oxidation states and multivalency of transition metals allow them to combine with a myriad of different organic and inorganic ligands, with coordination numbers from 2 to as high as 15,^19^ forming highly diverse three-dimensional structures. This opens an entire realm of chemical space that is not accessible to carbon-based scaffolds and provides the ″escape from flatland″ advocated by some medicinal chemists: a higher three-dimensional character correlates with higher clinical success rates.^20,21^ The superior geometrical diversity of metal complexes was recently demonstrated with a small library of 71 metallofragments (metal complexes with fragment-like ligands) that were shown to cover more three-dimensional chemical space than a representative organic fragment-library containing 18,000 molecules.^22^

The second unique characteristic of metal complexes is their ability to access multiple different and unique modes of actions. These range from redox reactions, generation of reactive oxygen species or catalytic generation of other active species, ligand exchange, or triggered ligand release. Different metals, and different types of metal complexes, are likely to act via widely varying, and potentially multiple, mechanisms. Overall, there are many ways for metal-based drugs to make a significant difference in the field of medicine and complement the organic drug arsenal currently available to us.^23^

While some metals, such as silver, have long been known to possess antimicrobial properties, there have been few systematic studies of anti-infective metal complexes until recently.^24−27^ In the last few years, several reports have described promising antibacterial properties of metal complexes,^28−31^ including our systematic study on the antimicrobial properties of ∼1000 metal-containing compounds contained within a screening collection of >300,000 molecules.^32^ These data were collected *via* the crowd-sourced Community for Open Antimicrobial Drug Discovery initiative (CO-ADD, co-add.org) funded by The Wellcome Trust and the University of Queensland. CO-ADD provides free antimicrobial screening to chemists around the world.^33^ Our analysis found that metal-containing complexes displayed significantly superior hit rates (9.9%) compared to purely organic molecules (0.87%), with cytotoxicity and hemolysis counter-screening assays showing similar toxicity rates for both classes, undermining the commonly held belief that metal-containing compounds are inherently (more) toxic.^32^

**Figure 1:**
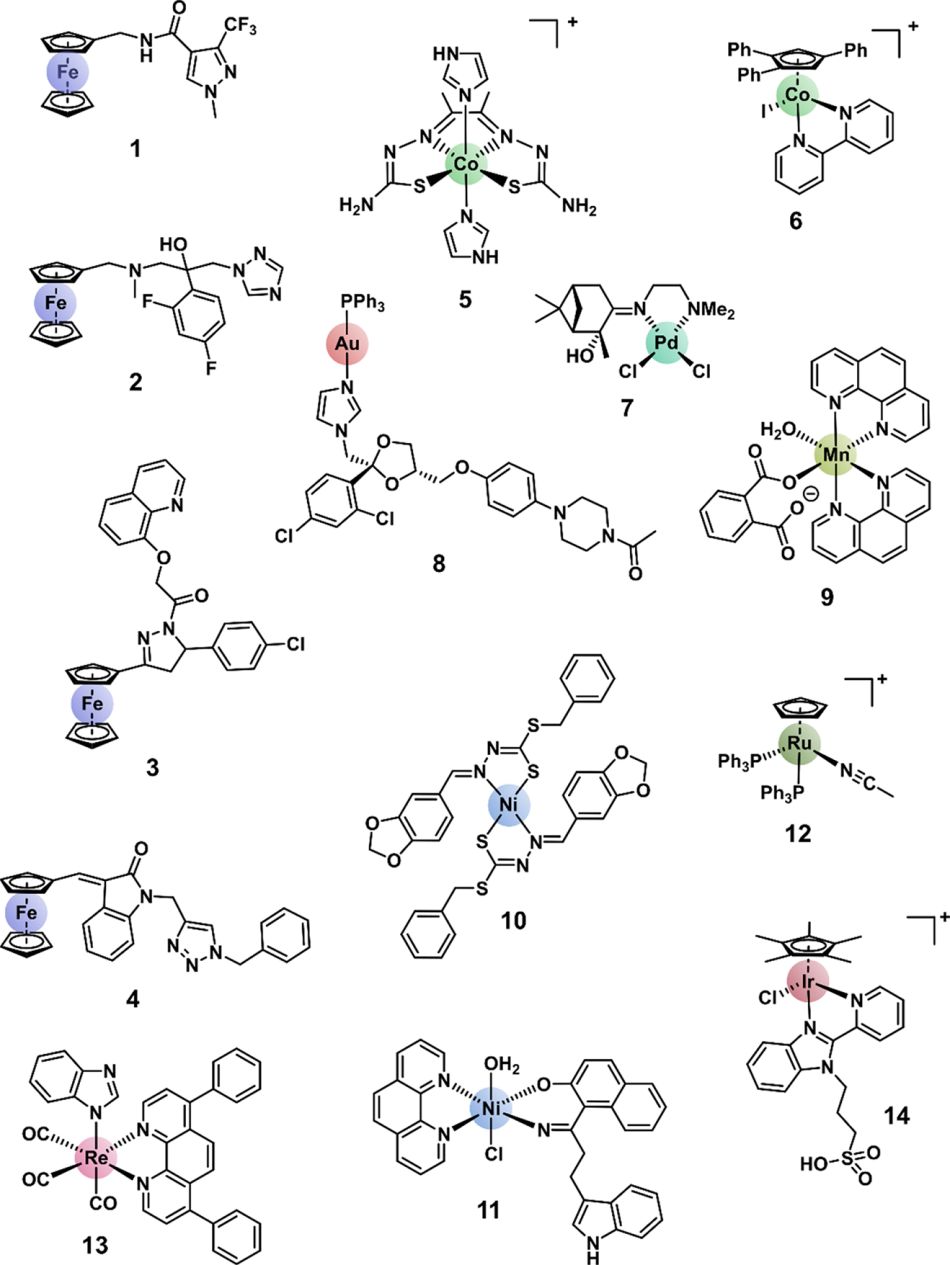
Selected structures of metal complexes with reported antifungal activity containing iron (**1,**^34,35^**2,**^35^**3,**^36^**4**^37^), cobalt (**5,**^28^**6**^38^), palladium (**7**^39^), gold (**8**^40^), manganese (**9**^41^), nickel (**10,**^42^**11**^43^), ruthenium (**12**^44^), rhenium (**13**^30^), and iridium (**14**^45^).

The antifungal activity of metal compounds has been investigated even less than their antibacterial properties.^28,30,34−51^Figure 1 presents an overview of selected metal compounds with measured antifungal activity. Gasser and coworkers recently published an expansive review of the metal-based antifungals research field.^52^ One issue that the authors noted, in addition to the small number of systematic studies into antifungal metal complexes, is the lack of mode of action studies and the often less than ideal antifungal data collection and reporting with regard to methodology and controls. Furthermore, there are only a few studies reporting *in vivo* evaluation of antifungal metal-based compounds.^28,30,35,51,53,54^ A series of phenanthroline complexes containing copper, manganese, or silver showed no toxicity in a *G. mellonella* (moth larvae) *in vivo* model at 10 μg/larva, and most complexes reduced the fungal burden of larvae infected with *Candida haemulonii*.^41,53^ Silver complexes of 1,10-phenanthroline-5,6-dione protected *G. mellonella* larvae from infection with *Phialophora verrucose*.^51^ A series of cobalt complexes (e.g., **5**, Figure 1) showed excellent *in vitro* activity against several fungal strains and displayed no toxicity in a *G. mellonellain vivo* model at doses up to 266 mg/kg.^28^ In 2021, a ferrocene-bearing fluconazole derivative with excellent *in vitro* antifungal activity (**2**, Figure 1) was evaluated in an *in vivo* mouse *Candida* infection model, where it both reduced the fungal burden and improved the inflammatory pathology of the mouse’s kidney and colon.^35^ The same year, rhenium complexes active against Gram-positive bacteria and fungi were tested for toxicity *via* a series of *in vitro* and *in vivo* assays, with the most promising complexes (e.g., **13**, Figure 1) showing no signs of cardio-, hepato-, or hematotoxicity or teratogenicity and inhibiting fungal filamentation in a *C. albicans* infection study in zebrafish.^30^

While these reports are encouraging, more systematic studies into metal-based antifungals are needed. The CO-ADD screening panel, while focused on five bacteria, also includes the pathogenic yeasts *Candida albicans* and *Cryptococcus neoformans*. Hence, we have access to an unprecedented array of systematically collected data on the antifungal properties of >300,000 compounds, including >1000 metal compounds. We now report on a large number of active and low-toxicity metal complexes identified during the screening of our library, with the most active ones subsequently tested against an extended panel of relevant fungal strains. A selection of compounds with the best activity profile was then evaluated for toxicity in the *in vivo* insect model *G. mellonella*, with most compounds exhibiting no toxicity at the tested concentrations. Finally, two nontoxic antifungal metal compounds were assessed in an *in vivoG. mellonella* efficacy (infection model) assay.

## Results and Discussion

As in our previous work, our use of the term ″metal complex″ refers to compounds containing d-block elements and lanthanides, as well as the post-transition metals gallium, indium, tin, thallium, lead, and bismuth. Actinides, the d-elements beyond atomic number 100, and the radioactive elements technetium and promethium are excluded. As of June 2020, a total of 1039 metal-containing compounds had been received and tested by CO-ADD. These compounds were submitted by 50 different research groups from 17 countries (and cover 32 of the 49 possible metal elements). The overall elemental distribution of the compounds has not changed significantly since our 2020 report. The most represented element is copper, with 187 compounds submitted to date.

Compounds were submitted to CO-ADD as dry powders, confirmed to be at >95% purity by the collaborators, and then tested as received. Characterization of the complexes were carried out by the submitting research group. No quality control check of purity was performed by CO-ADD due to the volume of compounds received. False positives are therefore possible, and promising compounds should be checked thoroughly before further development. The procedure reported in our previous study for antimicrobial testing by CO-ADD has been followed.^32^

**Figure 2:**
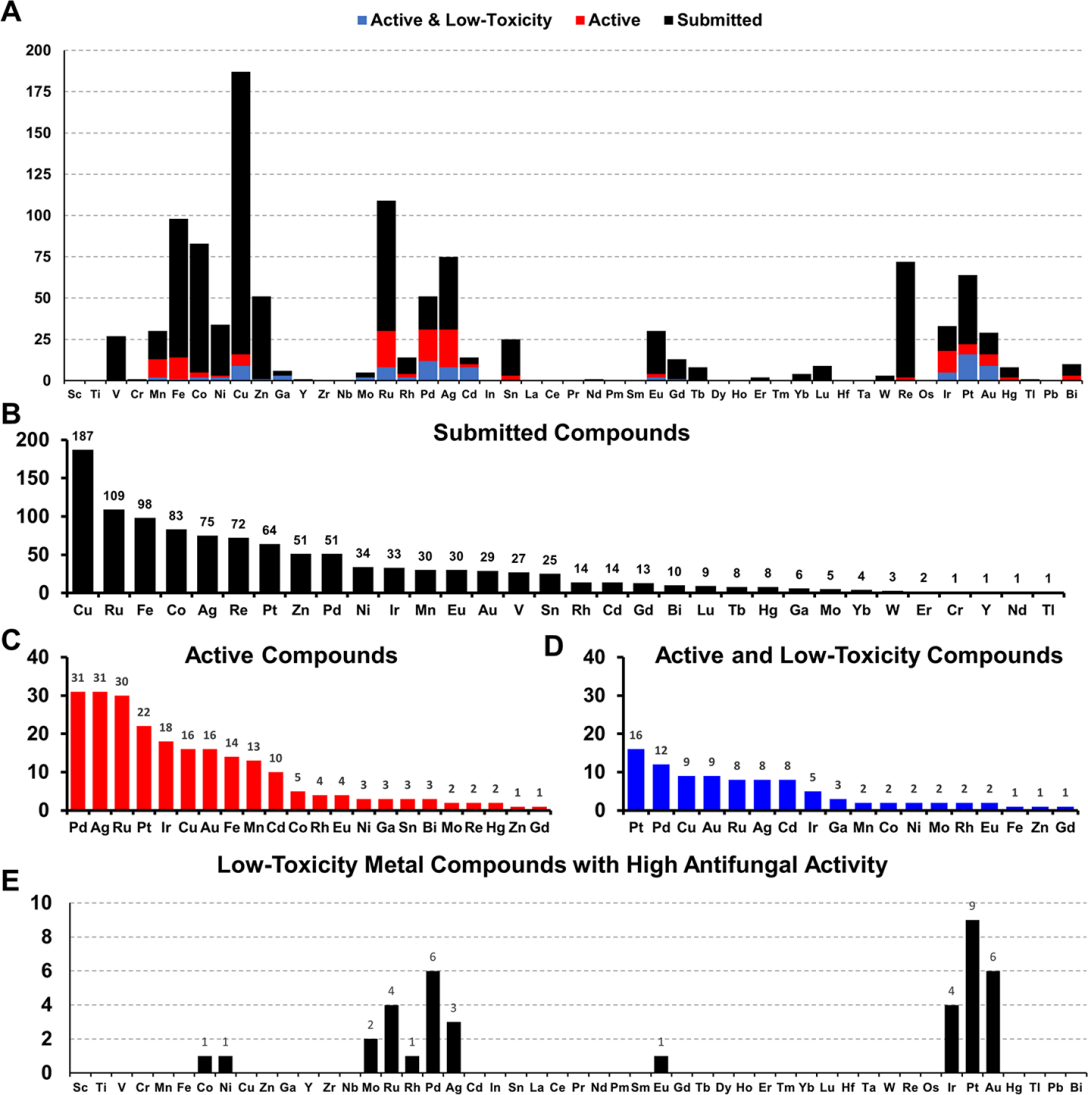
(A) Elemental distribution for all 1039 metal-containing compounds submitted to CO-ADD (black); 218/1039 submitted metal-containing compounds with at least one MIC lower or equal to 16 μg mL^–1^ or 10 μM (red) against the tested fungal organisms; 90/218 metal-containing compounds with antifungal activity and no cytotoxicity or hemolytic activity at the highest concentration tested (blue). (B) Metal frequency among the 1039 metal-containing compounds submitted to CO-ADD. (C) Metal frequency among the 218 metal complexes that possess some activity against the tested fungal organisms. (D) Metal frequency among the 90 compounds that are active against fungi as well as ″low-toxicity″ (see text for definition). (E) Elemental distribution of the 36 metal compounds (including two di-nuclear compounds) with high activity against the tested fungal strains, i.e., at least one MIC lower or equal to 2 μg mL^–1^ or 1.25 μM.

Of the 1039 tested metal compounds (Figure 2A,B), 320 (30.8%) showed activity against a bacterial and/or fungal strain (MIC ≤32 μg/mL). Two hundred eighteen (21.0%, Figure 2C) compounds had at least one MIC value that was less than or equal to 16 μg/mL or 10 μM against a fungal strain. Of these 218 antifungal metal compounds, 90 (10.4%, Figure 2D) complexes showed no cytotoxicity against HEK293 (human embryonic kidney) mammalian cells or hemolysis against human red blood cells up to 32 μg/mL or 20 μM. ″Low-toxicity″ compounds were defined as compounds with HEK293 CC_50_ >32 μg/mL or >20 μM and hemolytic HC_10_ >32 μg/mL or >20 μM (HC_10_ is the concentration causing 10% hemolysis).

The number of metal complexes with antifungal activity and low toxicity was further reduced by including only compounds with ″high″ antifungal activity, which was defined as having at least one MIC lower or equal to 2 μg/mL or 1.25 μM. This reduced the data set to 36 metal complexes (Figure 2E). It is worth noting that obtaining 36/1039 (3.5%) compounds with high antifungal activity and low toxicity from a crowd-sourced screen is a remarkable outcome. For comparison purposes, of the 287,534 small organic molecules for which an MIC was determined through CO-ADD against at least one fungal strain, only 3076 (1.1%) were found to be active. About half of these were further classified as toxic, leaving 1586 (0.6%) small organic molecules with activity against fungi and no toxicity at the measured concentrations. Applying the same ″high-activity″ filter to these compounds leaves 409 highly active ″low-toxicity″ organic molecules or 0.14% of all compounds tested, a 25-fold lower ″hit rate″.

**Figure 3:**
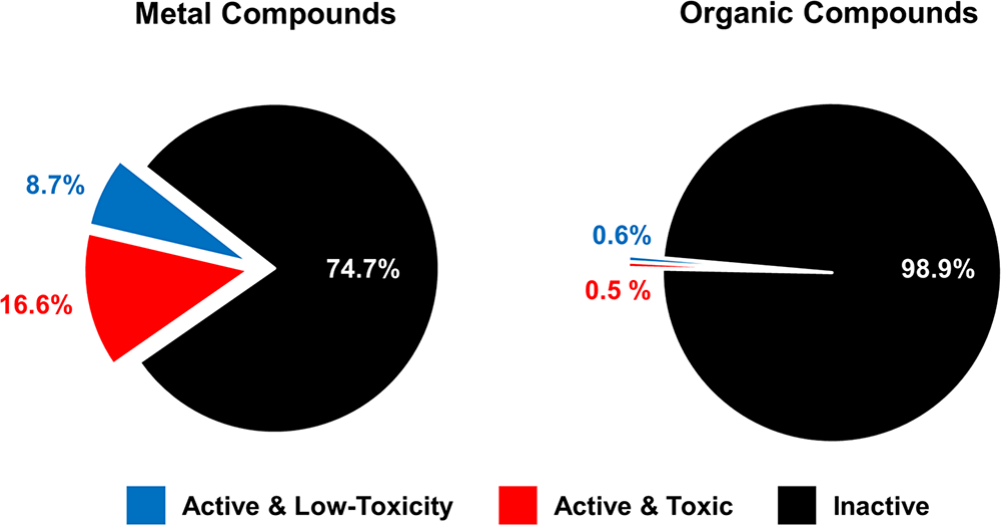
Percentage of submitted metal-containing compounds with antifungal activity with or without associated cytotoxicity and/or hemolysis compared to the overall hit rate for organic small molecules within the CO-ADD collection.

Two observations stand out when comparing the data for organic molecules with those for metal complexes. First, the overall antifungal hit rate for metal complexes is 23 times higher than that for small organic molecules, reconfirming our previous finding that metal compounds have superior hit rates against microbial organisms (Figure 3). The caveat is that the (metal) compounds tested by CO-ADD are not randomly selected. While some collaborators may have submitted compounds that are expected to have biological activities (e.g., against cancer), many compounds submitted to CO-ADD were originally made with very different applications in mind (*vide infra*).

When all the compounds displaying cytotoxicity are removed, the metal complexes still show a vastly superior rate of active and low-toxicity compounds (8.7 vs 0.6%, Figure 3). It is notable that the toxicity rate in the class of metal compounds was somewhat higher than that within the organic molecule group; *i.e.*, 66% of active metal complexes also showed toxicity against mammalian cell lines and/or hemolysis, whereas this was the case for only 48% of organic molecules. This contrasts our analysis focused on antibacterial activity where both organic and metal compounds had similar toxicity rates.^32^

Comparison of the distribution of all submitted compounds (Figure 2A,B) with the distribution of the active and low-toxicity compounds (Figure 2C,D) reinforces a trend that was observed previously, namely, that the first-row transition metals seem to be vastly under-represented in the active and low-toxicity group. Indeed, first-row transition metals make up 47% of submitted compounds but only 21% of all active and low-toxicity ones. With the still limited number of complexes, it is too early to dismiss these metals for antifungal applications altogether, but the current trends certainly seem to favor second- and third-row d-elements.

There may be good chemical reasons for this. Complexes of first-row transition metals tend to be less kinetically stable than those of the second row and especially the third row of transition metals. Also, essential transition (d-block) metals (Mn, Fe, Co, Cu, Zn, and Mo) have metabolic pathways specifically designed to control their homeostasis (uptake, transport, storage, and usage), so they may become diverted into targets other than the desired ones in the infective organisms.

To further explore the properties of these compounds, we reached out to the contributors of the 36 high-activity hit compounds to obtain fresh samples for further testing. Through this effort, we were able to obtain new stocks for 21/36 (58%) of the compounds shipped to our laboratories (Table 1). This number is impressive since these compounds were originally submitted for testing over a range of 5 years and the requests for more samples were sent out at the height of the COVID-19 pandemic when many laboratories were shut down for extended amounts of time.

**Table 1:**
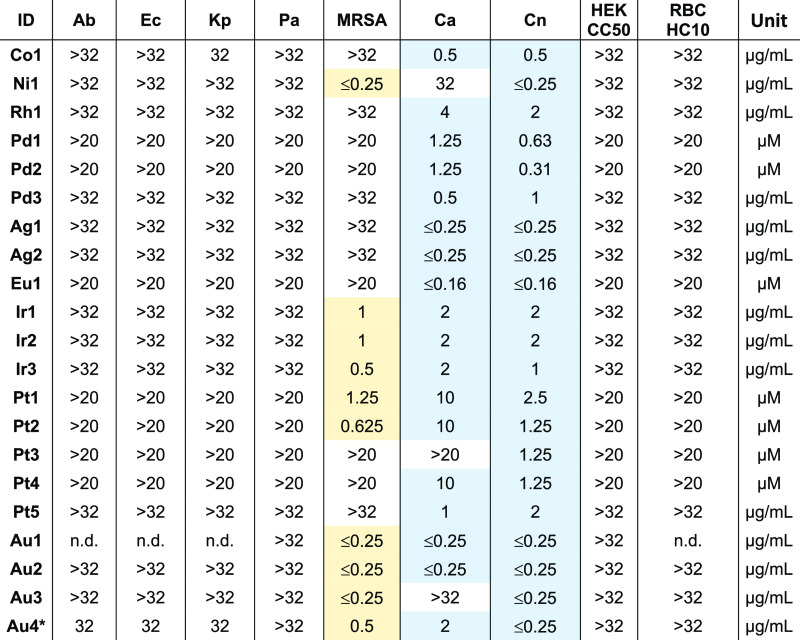
Initial CO-ADD Dose–Response Screening Data for 21 Metal Complexes with Potent Antifungal Activity and No Cell Toxicity up to 32 μg/mL or 20 μM, for which Fresh Stocks Could be Obtained (Values Given in μg/mL or μM Depending on How Compounds Were Originally Submitted to CO-ADD)^a^

a Ab, *Acinetobacter baumannii* ATCC 19606 type strain; Ec, *Escherichia coli* ATCC 25922 FDA control strain; Kp, *Klebsiella pneumoniae* ATCC 700603 ESBL; Pa, *Pseudomonas aeruginosa* ATCC 27853 QC control strain; MRSA, methicillin-resistant *Staphylococcus aureus* ATCC 43300; Ca, *Candida albicans* ATCC 90028 NCCLS11; Cn, *Cryptococcus neoformans* H99 ATCC 208821 type strain; HEK, HEK-293 human embryonic kidney cells ATCC CRL-1573; RBC, human red blood cells. N.d., not determined. Reference antifungal and antibiotics data provided in the Supporting Information.

b **Au4** was originally submitted to CO-ADD as a 1:2 mixture of **Au4a** and **Au4b**.

**Figure 4:**
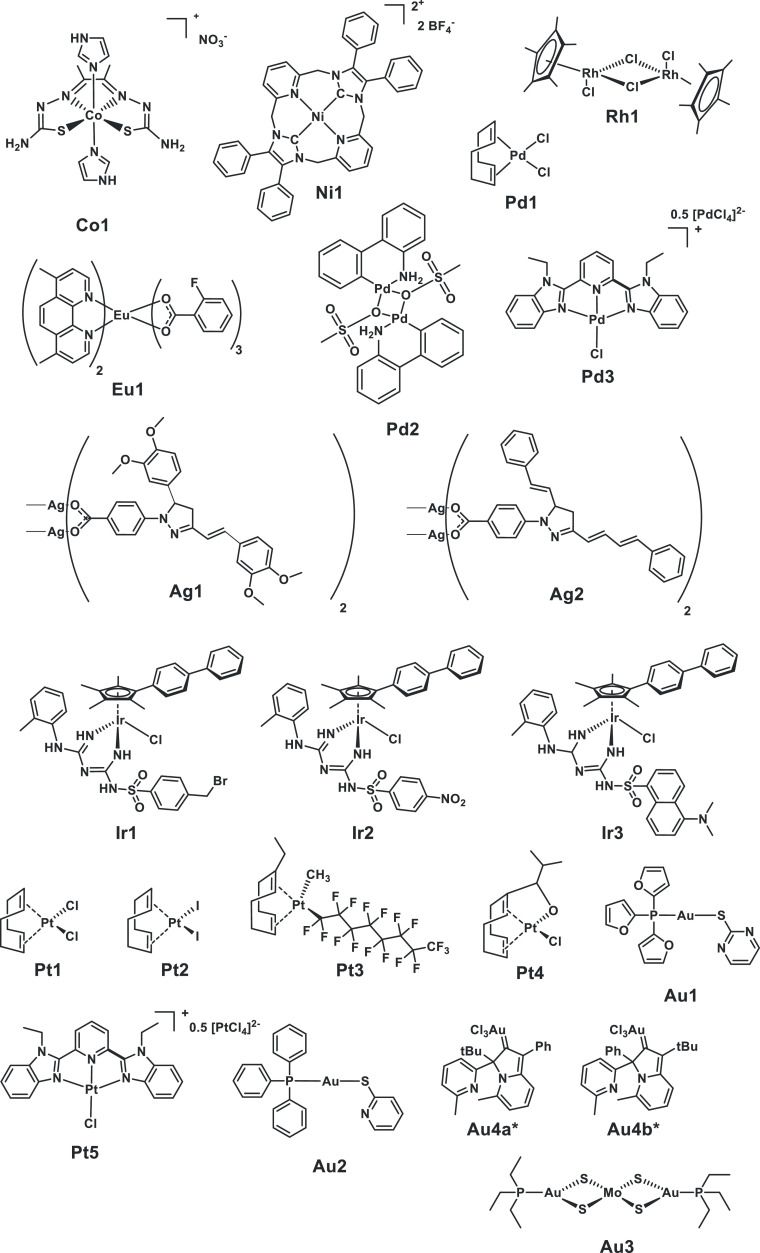
Chemical structures of the 21 metal complexes in the CO-ADD screening with high antifungal activity and no associated cytotoxicity and hemolysis. *Au4 is a mixture of two isomers (**Au4a** and **Au4b**); several samples were submitted with differing ratios.

The structures of the 21 obtained highly active metal complexes are shown in Figure 4. They comprise the nine elements cobalt(III), nickel(II), rhodium(III), palladium(II), silver(I), europium(III), iridium(III), platinum(II), molybdenum(VI), gold(I), and gold(III), with ruthenium the only element for which we were not able to obtain a second sample. The majority of these compounds (15/21) have not previously been studied for their antifungal activity. Synthesis protocols and characterization data have been reported elsewhere or are provided in the Supporting Information (Table S13). Notably, only five of these compounds are present in both this analysis and contained within the 30 metal complexes highlighted for their antibacterial activity in our earlier work.^32^ This indicates that somewhat different features are required to obtain activity against bacteria vs fungi. At the same time, the elements cobalt, ruthenium, silver, europium, iridium, and platinum are well represented in both data sets. While this can partly be explained by the high submission rates for compounds containing some of these elements, it perhaps suggests a trend in the activity patterns of metal-containing compounds. Increasing the number and diversity of tested metal complexes will be the only way to further support or contradict this finding.

These 21 complexes were tested against an extended panel of eight *Candida* and *Cryptococcus* strains. We used the same panel for the extended testing of a series of cobalt complexes (including **Co1**) in an earlier study.^28^ The panel comprises strains with different resistance profiles, including clinical isolates that are resistant to multiple classes of antifungal drugs. We also repeated the cytotoxicity and hemolysis assays with the new batch of compounds to verify earlier results and measure any possible adverse effects at higher concentrations (up to at least 100 μM).

From the initial set of antimicrobial testing and filtering for good activity, we expected a high degree of activity from the obtained 21 metal compounds. Indeed, we were pleased to find that most of the complexes showed good to excellent activity against most of the tested strains in the extended panel (Table 2).

**Table 2:**
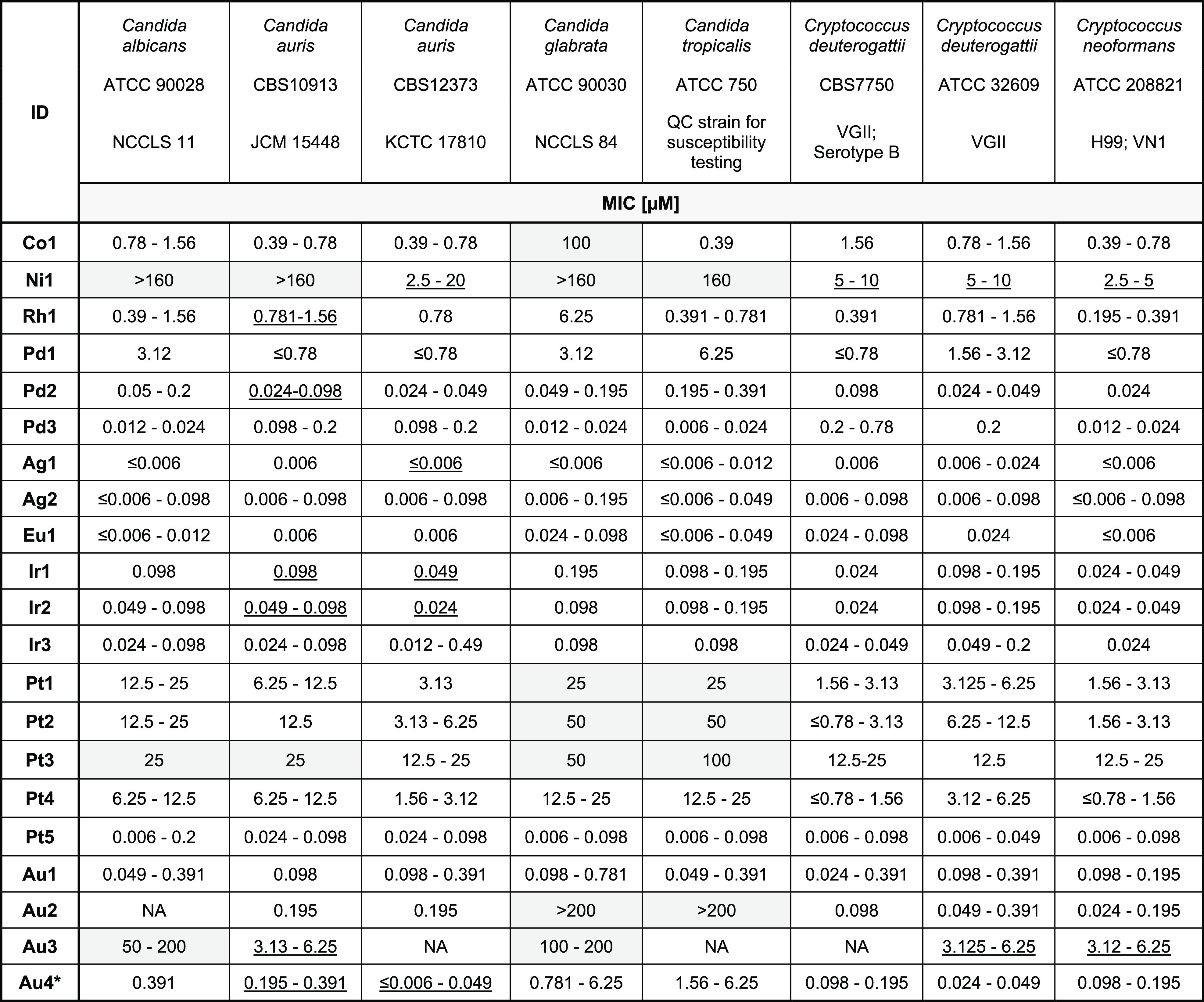
Extended MIC Testing against a Panel of Fungal Strains with Compounds for Which a Second Batch Could Be Obtained (MIC Is Displayed as ≥80 or ≥50% Inhibition in μM)^a^

a Underscored = MIC values that were inactive or gave a wide replicate variation when analyzed for ≥80% inhibition (optically clear to slightly hazy). These were re-analyzed at 50% inhibition (e.g., an MIC score of 2 = prominent decrease in visible growth as per CLSI M27 guidelines for evaluating MICs of yeasts) which gave more consistent and active values; see Table S9. NA: MIC value not available due to wide replicate variation.

b Two new samples of **Au4** (1:2 and 1:0.7, **Au4a**/**Au4b**) were received for further testing. Both mixtures gave the same MIC values across all assays.

As mentioned above, **Co1** is part of a series of Schiff-base complexes that were previously explored for both their anticancer^55,56^ and antifungal^28^ properties. The compound showed high levels of antifungal activity across the range of strains, with the notable exception of *Candida glabrata*. Importantly, we found no cytotoxicity or hemolysis up to the highest measured concentrations.

The nickel carbene complex **Ni1** was originally prepared to investigate the effect of structural changes upon the properties of N-heterocyclic carbene (NHC) complexes as catalysts for the electrochemical reduction of carbon dioxide, illustrating how compounds submitted were originally synthesized for a range of reasons. The first compound of this class was reported in 2004,^57^ with some more described in the recent work by Su *et al*.^58^ In the initial CO-ADD screening, this complex showed high activity against *Cryptococcus* spp. and the Gram-positive bacteria methicillin-resistant *Staphylococcus aureus* (MRSA) but none against the *Candida* spp. or any of the Gram-negative strains tested (Table 1). In the extended fungal panel, **Ni1** showed good activity against all the *Cryptococcus* spp. but no measurable effect against the *Candida* strains except *C. auris*. The complex also showed no toxicity or hemolysis up to the highest concentration measured, 160 μM. This heterogeneity in the fungal activity profile of this compound is notable as it is the only complex in this data set that shows this behavior. Culture media contributions can be ruled out as a factor as all tested yeasts were grown under the same conditions. Further studies on analogues of **Ni1** to investigate the origin of this *Cryptococcus* selectivity could lead to interesting, targeted antifungal candidates.

Rhodium complex **Rh1** is a useful synthon for the synthesis of rhodium piano-stool complexes.^59^ This compound showed high levels of activity against the entire panel of fungi, with MIC values in the nanomolar range and no cytotoxicity up to 200 μM. However, we found significant hemolysis in our second round of assays (Table S7 in SI), which was not observed in the initial CO-ADD screening. Previous studies of **Rh1** showed cytotoxicity against the human ovarian carcinoma cell line A2780 after prolonged (96 h) exposure (IC_50_ = 7.3 ± 1.5 μM).^60^ The strong hemolytic effect of **Rh1** precludes it from further testing.

Compounds **Pd1**, **Pd2**, and **Pd3** provide examples of the different structures that can be obtained within the chemical space of the same metal, oxidation state, and coordination geometry. All three square-planar palladium(II) complexes showed exclusively antifungal activity in the initial CO-ADD screening. The cyclooctadiene compound **Pd1** was part of a series of similar platinum complexes that we studied for their antibacterial potency, with some also showing antifungal activity (*vide infra*).^61^ Although **Pd1** showed good antifungal activity across all tested strains and no cytotoxicity up to 100 μM, it caused hemolysis at low concentrations, with a therapeutic index of 13. **Pd2** is a synthon for palladium catalysts.^62^ High levels of activity across the fungal panel were observed, with MIC values in the low nanomolar range, but were accompanied by some cytotoxicity and significant hemolysis (Table S7 in SI). Palladium complexes with ligands similar to **Pd3** have found some applications as catalysts for Suzuki-type reactions,^63^ while the ligand has been explored in other coordination complexes formed with lanthanides,^64−66^ cobalt,^67^ ruthenium,^68,71^ platinum,^69^ and chromium.^70^

Of note, compound **Pt5** is identical to **Pd3** in all aspects except the platinum metal center. Indeed, there are a total of nine complexes in the CO-ADD database with very similar structures, as well as the free ligand. The latter showed no activity at all in the CO-ADD primary screening and was not evaluated further. The biological evaluation of these analogue compounds has been reported separately.^72,73^ The fact that both **Pd3** and **Pt5** have similar activity profiles against the fungal panel but the free ligand alone shows no activity suggests that a metal is essential to obtain the observed antifungal activity. The cytotoxic and hemolytic properties of the two compounds are slightly different, with **Pd3** displaying low cytotoxicity and significant hemolysis. On the other hand, no hemolysis up to 200 μM could be detected for **Pt5**, and the cytotoxicity was similar to **Pd3,** resulting in a promising therapeutic index of >2500 (Table S7 in SI).

Silver compounds, silver ions, and silver nanoparticles have all been shown to be good antibacterial agents,^74−78^ but there have been comparatively very few studies into their antifungal properties,^79−81^ mostly focusing on silver nanoparticles.^82,83^ Compounds **Ag1** and **Ag2** are interesting as they show exclusive antifungal activity, with no inhibition of bacterial growth, suggesting that the activity is not due to free (weakly bound) silver ions; e.g., AgNO_3_ effectively inhibits both bacteria and fungi but also causes some degree of hemolysis in our assays (Table S2 in SI). The ligands for these complexes (**Ligand-Ag1** and **Ligand-Ag2** in the SI) were originally studied for their solvatochromism and were later coordinated with silver to investigate their potential antimicrobial activity.^84^ The carboxylate ion is able to coordinate to silver with monodentate, chelating, and/or bridging modes. Since the difference in frequencies (Δ*v*) between symmetric and asymmetric vibrations of the −COO group in FT-IR peaks is well under 200 cm^–1^, the bridging mode is suggested.^85,86^ The stability of complexes **Ag1** and **Ag2** was assessed. Solid samples kept at room temperature and protected from light exhibited identical NMR spectra over a period of 2 years. Complexes in DMSO solution also showed no changes in NMR (Figures S3 and S4 in SI) or UV–vis (Figure S5 in SI) spectra after 24 h.

Both silver compounds showed good antifungal activity with MICs ranging from 6 to 98 nM, but cytotoxicity CC_50_ values were in the low micromolar range (Table S7 in SI), though this still resulted in a good therapeutic index of 33,333 for **Ag2**. The free ligands of **Ag1** and **Ag2** were inactive (Table S2 in SI). **Ag1** and **Ag2** showed similar biological properties despite differing ligands, but a third silver complex with a very similar ligand showed significantly lower antifungal activity (**Ag-S4** in the SI). The antibacterial mode of action of silver ions has been studied in detail by the group of Sun *et al*.,^87,88^ but the antifungal mode of action remains unknown. Some reports have indicated that silver nanoparticles exert their antifungal activity through disruption of the cellular envelope, causing plasma membrane damage with subsequent cell leakage.^83,89^ It is noteworthy that *C. albicans*, but not *C. parapsilosis*, *C. tropicalis*, or *C. glabrata*, was shown to be able to convert Ag(I) ions into less toxic silver nanoparticles, thereby evading the antifungal effect of silver.^79^

Europium complexes have been studied for their versatile photophysical properties.^90−93^ There have been a few sparse reports on the potential antimicrobial properties of lanthanide complexes, including europium,^94^ and one europium complex with antibacterial activity was found in our previous analysis of CO-ADD data. A series of similar europium complexes (**Eu-S1**-**S8**, Figure S7 in the SI) are also in the CO-ADD collection. Several of these complexes show mild activity against Gram-positive MRSA and against the two tested fungal strains (Table S3 in SI). However, all but two complexes (**Eu-S1** and **Eu1**) also displayed cytotoxicity and/or hemolytic properties as well. The varied activity profile indicates a direct relationship between their structure and antimicrobial activity, toxicity, and hemolysis, suggesting that further optimization could lead to better compounds. In the extended panel, **Eu1** showed very high levels of activity against all the tested fungal strains (MIC values between 6 and 98 nM) and no cytotoxicity or hemolysis up to 200 μM, resulting in a therapeutic index of 33,333 (Table S7 in SI). Future studies could leverage the strong luminescence of this compound class for mode of action investigations.

The iridium complexes **Ir1–3**, with *N,N*-chelated ligands from the metformin (a biguanide anti-diabetic drug) family, were already highlighted in our previous antibacterial analysis for their ability to inhibit MRSA at low concentrations. Indeed, these complexes were already reported by the group of Sadler in an in-depth study on their antimicrobial properties.^95^ These three complexes maintained similar high levels of activity across the extended screening panel and exhibited moderate levels of cytotoxicity (CC_50_ values in the 50–100 μM range) with significant hemolysis (Table S7 in SI). Overall, the favorable therapeutic indices of 2600–3383 for **Ir1–3** are promising.

Compounds **Pt1**–**Pt4** are part of a series of cyclooctadiene compounds that were discovered through CO-ADD to have quite potent antibacterial activity,^61^ with some modest antifungal activity now reported. Compounds **Pt1**, **Pt2**, and **Pt4** were more active against the three *Cryptococcus* strains compared to the *Candida* strains. Promisingly, none of the four compounds showed any cytotoxicity or hemolysis up to 100 μM.

The antimicrobial properties of gold were first described by Robert Koch back in 1890, when he reported on the activity of potassium dicyanidoaurate(I) against *Mycobacterium tuberculosis*.^96^ More recent reports on the antimicrobial properties of gold complexes have been summarized in several review articles.^97−99^ The FDA-approved oral antirheumatic gold(I) drug auranofin exhibits excellent antibacterial properties *in vitro* and *in* vivo.^100,101^ Compounds **Au1** and **Au2** were originally part of a series of gold(I) phosphine thiolate complexes synthesized to investigate their biological properties.^102^ In the initial CO-ADD screening, where six other analogues were also tested (**Au-S1**-**S6**, Figure S8 in the SI), the two compounds stand out for their high activity against the two fungal strains as well as MRSA. The drastic change in activity profile with only a few atoms of difference between the compounds (Table S4 in SI) implies that both metal and ligands are responsible for the observed activity. For example, combining 2-sulfanylpyrimidine with a trifuran-2-yl-phosphane ligand gave complex **Au1** that had high activity against both MRSA and the fungal strains. In contrast, the gold complex with the same 2-sulfanylpyrimidine ligand but with triphenylphosphine (**Au-S4**, Figure S8 in SI) showed no activity against any of the tested strains. Conversely, the triphenylphosphine gold complex with a 2-sulfanylpyridine ligand (**Au2**) had high activity against MRSA and the fungal strains, but the same thiolate ligand with the trifuran-2-yl-phosphane gold complex (**Au-S1**) possessed only moderate activity. Interestingly, the introduction of a trifluoromethyl group on the thiolate ligand seemed to increase specificity toward bacteria, as no antifungal activity was observed for these compounds (**Au-S3** and **Au-S6**). In the extended panel, some slight differences in the activity patterns of **Au1** and **Au2** appeared. While **Au1** showed high antifungal activity against all fungal strains, **Au2** was not active against the *C. glabrata* and *C. tropicalis* strains. While the compounds showed only low to no hemolysis, they both displayed significant cytotoxicity in the repeated assays.

Complex **Au3** is perhaps the structurally most intriguing compound that we report in this study. Its synthesis was first described by Kinsch and Stephan in 1985,^103^ and it is the only heteronuclear bimetallic compound in this set of highly antifungal metal complexes. It is also the only one of five CO-ADD compounds that contains the element molybdenum, as Mo(VI). Notably, the related compound tetrathiomolybdate is currently in phase III clinical trials as a decoppering agent.^104,105^ All five molybdenum-containing compounds are of similar structures (**Au-S7**-**S10**, Figure S5 in SI), two containing phosphine ligands (**Au3** and **Au-S7**) and three with NHC ligands (**Au-S8**-**S10**). Again, we observed that depending on the exact structure of the compound, the observed activity was markedly different. Switching from a triethylphosphine ligand to triphenylphosphine resulted in complete loss of activity. A similar effect was observed with the NHC ligand. Unfortunately, we were not able to obtain more of **Au-S10** for further studies at this stage. It is notable that **Au3** seemed to possess high activity against MRSA and the *C. neoformans* strain while being not very active against *C. albicans.* Conversely, **Au-S10** only showed activity against *C. albicans*. Unfortunately, with **Au3**, we obtained wide ranges of activity for several of the fungal strains. Consistently good activity was observed against *C. auris* as well as with two *Cryptococcus* strains. Promisingly, **Au3** showed no hemolysis up to 200 μM and CC_50_ values in the range of 139–170 μM (Table S7 in SI), resulting in a therapeutic index of 44. The low toxicity values combined with signs of structure-dictated activity profiles suggest that synthetic explorations could yield even better compounds of this class.

Lastly, **Au4** (Figure 4) was part of a series of complexes containing an intact bis(pyridyl)allene framework (formed with palladium(II), platinum(IV), and gold(III)), a vinyl-platinum(II) metallacycle, and a series of gold(I) and gold(III) carbenes formed by the nucleophilic attack of an allenic pyridine into the allene moiety (Figure S10 in SI). Allene-containing complexes have recently been reported as potential anticancer agents, among other activities.^106^ The free ligands (**Au-Ligand1** and **Au-Ligand2**), as well as the corresponding platinum(IV) complexes, did not show any antimicrobial activity (Table S6 in SI). The palladium(II) analogues showed good activity against MRSA and the two yeasts tested but were cytotoxic. The two gold(III) analogues with allene ligands coordinated through the pyridyl nitrogens (**Au-S11** and **Au-S12**) showed moderate to good activity against the two fungal strains tested but varied dramatically in hemolytic activity despite only varying by a phenyl to a *tert*-butyl group on the ligand framework. The vinyl-Pt(II) metallacycle derivative showed good activity against MRSA and the two yeasts tested but also exhibited high levels of cytotoxicity. This platinum complex and the novel gold carbenes series were originally studied for their application as potential catalysts for the cyclization of 1,6-enynes, and no biological activity for them has been reported to date.^107^

From this series, only the gold(III) carbene **Au-S13** showed some moderate activity against any of the tested Gram-negative strains as well as high activity against MRSA and yeasts. However, both **Au-S13** and its gold(I) analogue (**Au-S14**) showed high toxicity levels, leaving only the gold(I) complex **Au-S15** as an active and low-toxicity candidate. Unfortunately, we were unable to obtain more of it for further testing. Gold(III) complex **Au4** showed good levels of activity across the antifungal panel and no cytotoxicity or hemolysis up to 200 μM, resulting in a therapeutic index of 33,333 (Table S7 in SI). The original sample of **Au4** submitted to CO-ADD was received as a 1:2 mixture of nonseparable but well-characterized isomers (**Au4a**/**Au4b**). For the expanded panel, two new samples were obtained with ratios of 1:2 and 1:0.7 (**Au4a**/**Au4b**), respectively. Both samples showed virtually identical MIC values, indicating that both isomers are responsible for the observed activity.

### General Observations

Some overall observations can be made upon analysis of the results from the extended antifungal and toxicity assays. The level of activity for the most active compounds is comparable to the activity of the control antifungal drugs against the respective pathogen (Tables S10 and S11 in SI). And, generally, good activity in the initial CO-ADD screening translates to good activity across the whole panel of *Candida* and *Cryptococcus* strains used in this work.

The same cannot be said for the cytotoxicity and hemolysis counter-screening data, with significant variations often seen between different assay runs. Part of this can be attributed to the fact that the highest concentration measured in the initial CO-ADD screening is 32 μg/mL (standard units for antimicrobial screening), which, depending on the molecular weight of the compound, can vary dramatically when translated to molar units. Because the molecular weight of metal complexes generally covers an expanded range compared to organic compounds, all subsequent assays were conducted with molar units to generate comparable data. In essence, this work highlights that the cytotoxicity of these compounds needs to be evaluated rigorously. It should be noted that the therapeutic indices for most (14/21) compounds tested in the extended panel were still over 50 and many (9/21) were >1000, indicating the significant potential for further development (Table S7 in SI). As can be seen in this study, conducting all assays in the same laboratory and under the same conditions can still produce variable results. This underscores the need to standardize the testing conditions for metal-based antimicrobials to obtain reliable data and advance the field.

Based on criteria of overall antifungal activity, cytotoxicity, and hemolysis as well as compound availability, we selected compounds **Co1**, **Ag2**, **Eu1**, **Ir1**, **Pt1**, **Pt2**, **Pt4**, **Pt5**, **Au1**, **Au2**, and **Au4** to conduct a preliminary *in vivo* toxicity assay. For these initial *in vivo* assessments, we used the greater wax moth *G. mellonella* instead of a rodent model,^28,61^ as it is significantly less resource- and cost-intensive, allowing us to screen far more compounds than would otherwise be possible. Results obtained in *G. mellonella* have been shown to be robust, reproducible, and correlate well with toxicity study results obtained in rodent models.^108−110^ The compounds were dissolved in DMSO and diluted to the highest possible final concentration (Table 3). Larvae were injected with 10 μL of the compound (at the maximum concentration indicated in Table 3) or DMSO control. The larvae were then monitored for 6 days for survival and health using the *G. mellonella* Health Index Scoring System.^111^ Of the 11 tested compounds, only **Au2** showed signs of toxicity at the highest tested concentration (1 mM). For the other compounds, no detrimental effects could be observed in the larvae over the monitoring period. These data contribute to our understanding that metal complexes need not be considered as generally toxic. Nevertheless, factors such as the metabolism, excretion, and/or accumulation of the various metal complexes and their metabolites will still need to be studied more rigorously in rodent models if they are to be advanced into the preclinical pipeline. Of note, the maximum concentration reached in the larvae in these assays is anticipated to be significantly lower than the concentrations measured in the *in vitro* toxicity/hemolysis assays.

**Table 3:** Results of the *In Vivo* Toxicity Assay in *G. mellonella* (10 μL of the Highest Concentration Was Injected into the Larvae)

compound	max conc.	max dose	toxicity
**Co1**	10 mM	244 mg/kg	nontoxic
**Ag2**	1 mM	53 mg/kg	nontoxic
**Eu1**	1 mM	49 mg/kg	nontoxic
**Ir1**	1 mM	47 mg/kg	nontoxic
**Pt1**	0.4 mM	7 mg/kg	nontoxic
**Pt2**	0.4 mM	11 mg/kg	nontoxic
**Pt4**	0.4 mM	8 mg/kg	nontoxic
**Pt5**	1 mM	38 mg/kg	nontoxic
**Au1**	1 mM	27 mg/kg	nontoxic
**Au2**	1 mM	28 mg/kg	toxic at 1 mM
**Au4**^a^	1 mM	33 mg/kg	nontoxic

a For the *in vivo* testing, the **Au4** sample with a ratio of 1:0.7 (**Au4a**/**Au4b**) was used.

With these results in hand, we proceeded to conduct an exploratory *in vivo* efficacy study with compounds **Co1**, **Eu1**, **Ir1**, **Pt1**, **Pt2**, **Pt4**, **Au1**, and **Au4** (**Ag2** and **Pt5** had to be excluded due to solubility issues in these assays). In an initial survival assay, *G. mellonella* larvae were inoculated with *C. albicans* (ATCC 90028). The larvae were then injected with 10 μL of the compound solution, and their survival was monitored for the next 5 days. Fluconazole and 10% DMSO injections were used as controls. Compounds **Pt1** and **Pt2** and the fluconazole control resulted in significant larva survival (at least three larvae alive after 5 days at the tested concentrations) compared to the untreated control.

To quantify the possible protective effect of **Pt1** and **Pt2** in *G. mellonella* challenged with *C. albicans*, we conducted a CFU reduction assay with these compounds. Briefly, *G. mellonella* larvae were inoculated with *C. albicans* (ATCC 90028) and incubated for 2 h at 37 °C. The larvae were then injected with 10 μL of the corresponding compound solution (1 mM) or control (same concentration and injection volume) and incubated for 24 h at 37 °C. The larvae were then anesthetized, macerated, and serially diluted onto SDA (Sabouraud dextrose agar) plates. After 48 h of incubation, the CFUs were counted.

**Figure 5:**
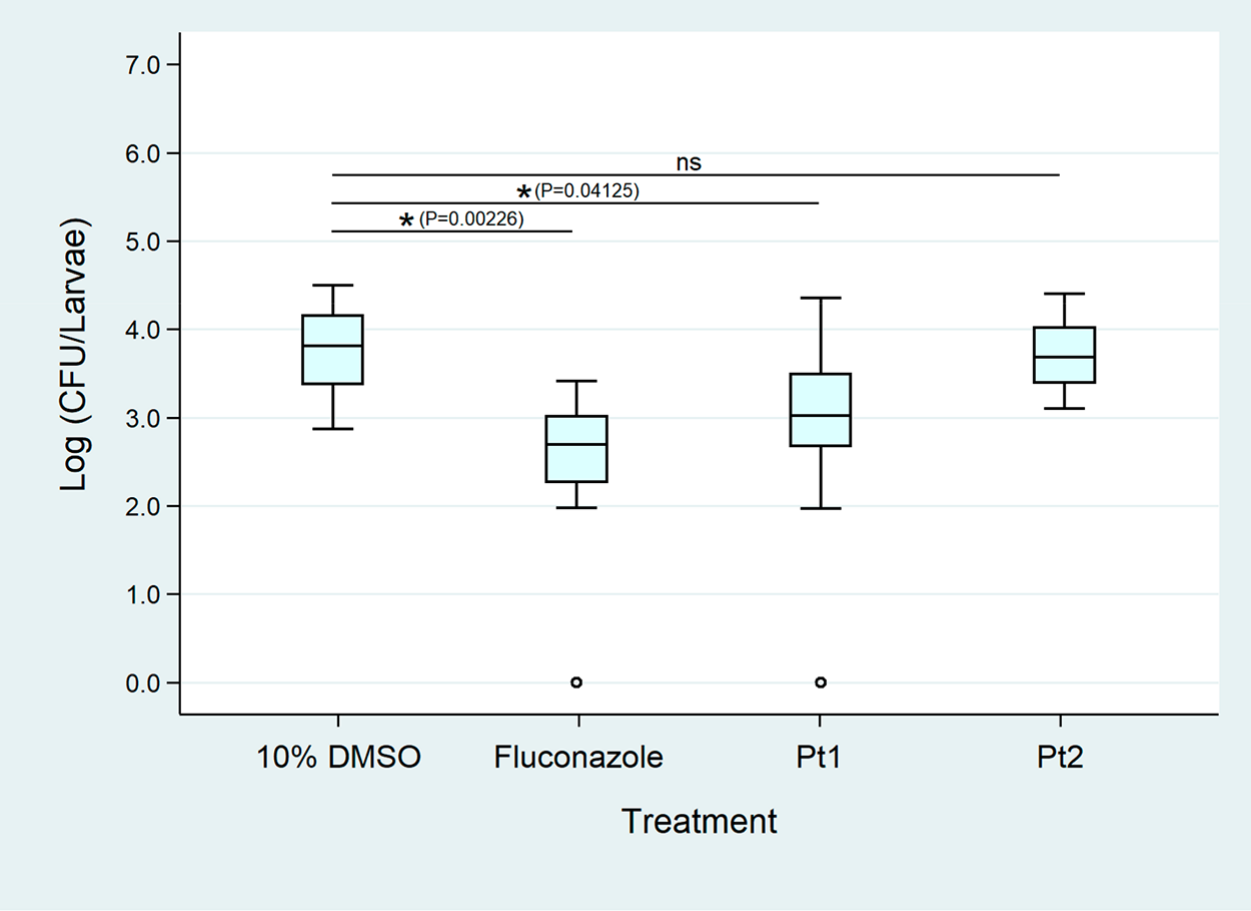
Average weight-standardized log10 CFU counts for *G. mellonella* larvae challenged with *C. albicans* and corresponding compounds **Pt1** (19 mg/kg), **Pt2** (28 mg/kg), and the controls, fluconazole (12 mg/kg) and 10% DMSO, after 24 h incubation at 37 °C. Statistical significance calculated by one-way ANOVA analysis with Tukey’s pairwise test: ns = not significant.

**Pt2**-treated larvae showed CFUs comparable to the DMSO control, indicating that no significant biological effect was achieved by this compound at the tested concentration (Figure 5). On the other hand, a significant reduction in CFUs was detected for the control compound fluconazole (1.1 log10) and for compound **Pt1** (0.7 log10). These data indicate that **Pt1** is able to reduce the fungal burden in a living organism. Together with the data on its promising antibacterial properties, **Pt1** and its related compounds seem to be a promising starting point for a novel class of antimicrobial agents.

## Conclusions

This work represents the first large-scale investigation of metal complexes as antifungal agents. Through CO-ADD, over 1000 metal-containing compounds submitted by a range of research groups were tested for their antifungal activity against *C. albicans* and *C. neoformans*. Similar to our analysis of data for antibacterial properties,^32^ we found that metal complexes had a significantly higher (23×) hit rate against these fungal strains when compared with the over 300,000 tested organic molecules. These data should still be regarded as preliminary since the number of metal complexes tested is still relatively low, particularly if the immense chemical space accessible through all possible metal–ligand combinations is considered. Nevertheless, this study makes a strong case that a more systematic and thorough study of metal-based compounds for antimicrobial applications is warranted.

In our previous antibacterial study, both metal compounds and organic molecules displayed roughly equal rates of cytotoxicity and hemolysis (around two-thirds of all tested compounds displayed toxicity at the tested concentrations). In the current antifungal analysis, we found that the incidence of toxicity for metal complexes (66%) was moderately elevated compared to the organic molecules tested (48%). It should be noted that the toxicity rate for metal compounds was very similar to that reported in our earlier study, whereas the rate for organic molecules was reduced. However, even with their relatively higher toxicity rate, the overall percentage of metal complexes that were active against the tested fungi yet exhibited low toxicity was still significantly higher than their organic counterparts (8.7 vs 0.6%).

To further evaluate the potential of these metal complexes, we obtained fresh samples for 21 of the most promising compounds and assessed their antifungal potential against a panel of eight relevant fungi. We found that the high activity observed in the initial assay against *C. albicans* and *C. neoformans* generally translated to high activity against the broader panel. We also studied the potential cytotoxicity and hemolysis of these compounds at higher concentrations and found that, in several cases, some toxicity or hemolysis was now observed. Wishing to understand the potential toxicity of metal compounds in living systems, where they have been assessed far less often than organic drug candidates, we tested 11 of these active metal complexes in a *G. mellonella* larva toxicity model. Ten exhibited no toxicity at the highest injected concentration. These data, together with results from other recent studies, indicate that metal complexes in general are well tolerated by the *G. mellonella* model.^14,28,61,112^

Compounds submitted to CO-ADD are generally not optimized for biological applications; hence, any hits obtained through this screening will most likely have to undergo medicinal chemistry optimization before a potential drug candidate is obtained. Regardless, we were interested to see if the excellent *in vitro* antifungal activity observed for metal complexes would translate to *in vivo* efficacy prior to any structural optimization for solubility, stability, and other properties. To this end, we evaluated the ability of eight metal complexes to prolong survival in *G. mellonella* larvae infected with *C. albicans.* Compounds **Pt1** and **Pt2** showed some efficacy, and treatment with **Pt1**, but not **Pt2**, resulted in a significant 0.7 log10 reduction in the treated larvae compared to the DMSO control. As mentioned earlier, we have previously reported on **Pt1** and related compounds for their activity against Gram-positive bacteria. Interestingly, in an analogous *in vivo* assay with *G. mellonella* infected with MRSA, compound **Pt1** did not elicit a significant reduction in bacterial load.^61^ It is notable that this compound class seems to perform well in assays against microbes while showing no toxicity against human cell lines or in *G. mellonella*.

These results warrant further studies into the structure–activity relationship of this compound class. Initial insights showed that substitution of the chloride ligands on **Pt1** generally results in a reduction of antibacterial activity, suggesting that chemical alterations of the COD-fragment may be more favorable.^61^ One concern with **Pt1** is its potential stability under biological conditions. In a preliminary stability study, incubation of **Pt1** in DMSO for 7 days at room temperature resulted in no changes of the ^1^H NMR spectrum, suggesting that the compound is stable under these conditions (Figure S6 in SI). Future work will be aimed toward more comprehensive examinations of the chemical and biological stability of these and related compounds, as well as investigations into their mode of action.

In summary, this study continues our efforts to showcase the vast potential of metal-containing compounds as antimicrobial agents. We have shown that metal complexes have promise as antifungal agents, displaying hit rates that vastly surpass those of a similarly sourced set of organic molecules. While metal compounds did show slightly higher rates of toxicity, they are overall still 14× times more likely to be active against *C. albicans* or *C. neoformans* in our data set. We further showed that this activity is generally retained against other fungal species and strains, including drug-resistant isolates. Lastly, we demonstrate that most of these metal complexes are well tolerated by *G. mellonella*, displaying no signs of toxicity, and we identify one compound, **Pt1**, with the ability to significantly reduce the load of *C. albicans* in a moth larva infection model.

The results also validate the hypothesis behind the founding of CO-ADD, i.e., that searching for new antimicrobials without excluding potential new chemotypes due to the many dogmas of drug discovery may help to refill the antibiotic pipeline. We do note that the selection of metal complexes tested is biased by the collaborator’s motive in making and submitting compounds, but the same caveat applies to submitted organic compounds. CO-ADD’s phenotypic screening approach, traditionally used for antimicrobial discovery, is currently undergoing a renaissance for other therapeutic areas.^113^

Together with our recent studies, this work strongly supports further investigations into metal complexes as potential antimicrobial agents. We encourage other researchers to conduct more extensive studies into these compound classes. In particular, future work should focus on more systematic structure–activity relationship studies as well as elucidating the mode of action of active metal compounds. The few in-depth studies on the mechanism of action of metallobiotics in recent years have indicated that metal compounds are likely to affect multiple targets inside of microbes.^17,26,114,115^ This makes them well suited to avoid rapid resistance development but also increases the difficulty in narrowing down their exact mode of action.^116^ On behalf of CO-ADD, we invite researchers around the world to submit their (well-characterized) metal complexes for antimicrobial testing to advance our collective knowledge on this promising and underexplored compound class.

## Methods

### Purity of Compounds

All compounds were obtained as dried powders from collaborators and confirmed by the collaborators to be at >95% purity. No further purification was performed by CO-ADD. The dry compounds were dissolved to a final concentration of 10 mg/mL or 10 mM in DMSO and used for the screenings as such.

### Antibacterial Assays

For the all the bacterial assays, each bacterial strain was cultured in cation-adjusted Mueller Hinton broth (CAMHB; Bacto Laboratories 212322) at 37 °C overnight. A sample of each culture was then diluted 40-fold in fresh CAMHB and incubated at 37 °C for 1.5–3 h. The resultant mid-log phase cultures were diluted with CAMHB (CFU/mL measured by OD_600_) and then added to each well of the compound-containing plates (384-well non-binding surface (NBS) plates; Corning CLS3640), giving a cell density of 5 × 10^5^ CFU/mL and a total volume of 50 μL. Plates were covered and incubated at 37 °C for 18 h without shaking. Inhibition of bacterial growth was determined by measuring the absorbance at 600 nm (OD_600_) using media only as negative control and bacteria without inhibitors as positive control. MIC values were determined as the lowest concentration at which the growth was inhibited by ≥80% (equivalent to no visible growth by the eye). Colistin sulfate (Sigma C4461) and vancomycin HCl (Sigma 861987) were used as internal controls on each plate for Gram-negative and Gram-positive bacteria, respectively. All compounds were tested as two technical replicates in two independent biological assays, *n* = 4 final data.

### Antifungal Assays

For the fungal assays, both fungi (yeast) strains were cultured for 3 days on Yeast Extract-Peptone Dextrose (YPD; Becton Dickinson 242720) agar at 30 °C. A yeast suspension of 1 × 10^6^ to 5 × 10^6^ CFU/mL (as determined by OD_530_) was prepared from five colonies from the agar plates, subsequently diluted with Yeast Nitrogen Base media (YNB; Becton Dickinson 233520), and added to each well of the compound containing plates (384-well plates, NBS; Corning CLS3640), giving a final cell density of 2.5 × 10^3^ CFU/mL and a total volume of 50 μL. Plates were covered and incubated at 35 °C for 36 h without shaking. The growth inhibition of *C. albicans* was determined by measuring the absorbance at 630 nm (OD_630_), while the growth inhibition of *C. neoformans* was determined by measuring the difference in the absorbance between 600 and 570 nm (OD_600–570_), after the addition of resazurin (0.001% final concentration; Sigma R7017) and incubation at 35 °C for 2 h, using media only as negative control and fungi without inhibitors as positive control. MIC values were recorded as the lowest concentration at which the growth was inhibited by ≥80%, equivalent to ″optically clear-to-slightly hazy″ or MIC score of 0–1 as per CLSI guidelines M27 Reference Method for Broth Dilution Antifungal Susceptibility Testing of Yeasts. Using this same scoring method, MICs were calculated at score 2 or 50% inhibition, with results displayed in the Supporting Information. Fluconazole (Sigma F8929) was used as internal control on each plate for all strains. All compounds were tested as two technical replicates in two to four independent biological assays, *n* = 4–8 final data. Those compounds that showed greater variability in data were tested at the higher replicate number.

### Cytotoxicity Assays

HEK-293 ATCC CRL-1573 human embryonic kidney cells were counted manually in a Neubauer hemocytometer and added to compound-containing plates (384-well plates, tissue culture treated (TC); Corning CLS3712), giving a final density of 5000 cells/well and a total volume of 50 μL, using Dulbecco’s modified Eagle medium (DMEM; Life Technologies 11995-073) with 10% fetal bovine serum (FBS; GE SH30084.03). The cells were incubated together with the compounds for 20 h at 37 °C in 5% CO_2_. Cytotoxicity (or cell viability) was measured by fluorescence, ex: 560/10 nm, em: 590/10 nm (F560/590), after addition of 5 μL of 25 μg/mL resazurin (2.3 μg/mL final concentration; Sigma R7017) and after further incubation for 3 h at 37 °C in 5% CO_2_, using media only as negative control and cells without inhibitors as positive control. CC_50_ (concentration at 50% cytotoxicity) values were calculated by curve fitting the inhibition values vs log(concentration) using a sigmoidal dose–response function, with variable fitting values for the bottom, top, and slope. Tamoxifen (Sigma T5648) was used as internal control on each plate.

### Hemolysis Assays

Human whole blood (Australian Red Cross) was washed three times with 3 vol of 0.9% NaCl and resuspended in a concentration of 0.5 × 10^8^ cells/mL, determined by a manual cell count in a Neubauer hemocytometer. Washed cells were added to compound-containing plates (384-well polypropylene plates (PP); Corning 3657) for a final volume of 50 μL, shaken, and incubated for 1 h at 37 °C. After incubation, the plates were centrifuged at 1000*g* for 10 min to pellet cells and debris; 25 μL of the supernatant was then transferred to reading plates (384-well, polystyrene plated (PS), Corning CLS3680), with hemolysis determined by measuring the supernatant absorbance at 405 mm (OD405) using cells without inhibitors as negative control and cells with 1% Triton X-100 (Sigma T8787) as positive control. HC10 and HC50 (concentration at 10 and 50% hemolysis, respectively) were calculated by curve fitting the inhibition values vs log(concentration) using a sigmoidal dose–response function with variable fitting values for the top, bottom, and slope. Melittin (Sigma M2272) was used as internal control on each plate. The use of human blood (sourced from the Australian Red Cross Blood Service) for hemolysis assays was approved by the University of Queensland Institutional Human Research Ethics Committee, Approval Number 2014000031.

### *Galleria mellonellaIn Vivo* Toxicity Assay

The toxicity of compounds was tested *in vivo* using the *G. mellonella* model using our previously described methods,^61^ Briefly, *G. mellonella* larvae were reared in a controlled environmental room at Macquarie University, Sydney, Australia, at 30 °C and 65% humidity with a 12-h light/dark cycle. Larvae (200–250 mg) were individually injected with 10 μL of chemical into the last right pro-leg using a 100 μL syringe (Hamilton Ltd.). Each compound was dissolved in DMSO at the maximum concentration given in Table 3 and also diluted in water to final concentrations of 100, 10, and 1 μM. We injected five larvae in triplicate for each of the four dilutions for each compound. Larvae injected with different dilutions of DMSO (10^–1^, 10^–2^, and 10^–3^) were included as negative controls. Following injection, the larvae were incubated at 30 °C and monitored every 24 h for 6 days. Larval performance was assessed according to the *G. mellonella* Health Index Scoring System.^111^ The experiments were repeated over three separate days for biological triplicates.

### *Galleria mellonella* Infection (Survival) Assay

The globally available strains for *C. albicans* and *C. neoformans*, namely, ATCC 90028 and H99, respectively, were used to assess the antifungal potential of the panel of cobalt complex compounds in the *G. mellonella* insect model. Each strain was cultured on Sabouraud dextrose agar (SDA) for 24–48 h at 27 °C prior to inoculation. Yeast colonies were then suspended in a phosphate-buffered saline solution (PBS), and cells’ concentrations were adjusted using a Neubauer counting chamber to 5 × 10^7^ cells for ATCC 90028 and 1 × 10^8^ cells for H99. Compounds were prepared by dissolving in 100% dimethylsulfoxide (DMSO) to 4 or 10 mM depending on compound solubility before being further diluted with water to 0.4 or 1 mM for injection into larvae.

For each compound, five sixth instar *G. mellonella* larvae of similar size (ranging from 200 to 250 mg) were selected for each fungal species and injected with 10 μL of the fungal inoculum in the last left pro-leg using a Hamilton (USA) 1710 TLL syringe with a 27-gauge needle. Each group of larvae was subsequently incubated at 37 °C for 2 h in 90 mm Petri dishes. After incubation, larvae were injected with 10 μL of the compound into the last right pro-leg and returned to Petri dishes to incubate at 37 °C for 5 days. In addition to the panel of metal compounds, fluconazole (FLC) was included as a positive control (1 and 0.4 mM) and reference antifungal. Larvae inoculated with just the fungal strains and the fungal strains with 10% DMSO were also included as comparison groups. Larvae injected concurrently with PBS instead of fungal inoculum and compounds were used as negative controls. All larvae were checked daily for survival.

### *Galleria mellonella* Infection (Log CFU Reduction) Assay

A log CFU reduction assay was conducted for two compounds and two control groups. The compounds examined were **Pt1** (1 mM) and **Pt2** (1 mM), both of which showed observed *in vivo* antifungal potential with the *G. mellonella* survival assay against the *C. albicans* strain ATCC 90028. Fluconazole (1 mM) and 10% DMSO were used once again as the control groups. Fifteen *G. mellonella* larvae (200–250 mg) per test group were selected and inoculated with 10 μL of *C. albicans* at a concentration of 5 × 10^7^ cells/mL and incubated on 90 mm Petri dishes in groups of five larvae for 2 h at 37 °C. Larvae were then subsequently injected with 10 μL of the corresponding test compound or control and returned to Petri dishes to incubate for 24 h at 37 °C.

Larvae were then anesthetized on ice for 5 to 10 min before being placed in individual prefilled 2 mL tubes with 3.0 mm diameter zirconium beads with 1000 μL of PBS. They were then macerated briefly using the BeadBug 6 Homogenizer (Benchmark Scientific, USA) in two 45 s intervals at 4350 rpm. After homogenization, serial dilutions of 1:100, 1:1000, and 1:10,000 were then made for each larva/tube, and 100 μL of the dilution was spread plated onto SDA plates with 50 μg/mL chloramphenicol. Plates were then incubated at 27 °C for 48 h before CFUs were counted.

A one-way ANOVA was conducted to compare the CFU counts for **Pt1**, **Pt2**, and both controls. After a significant difference was observed between the groups, a subsequent Tukey’s pairwise test was used to compare the individual test groups with each other.

## Supporting Information Available

Experimental details, characterization data, and additional data (PDF)

## Notes

An excellent review on the emerging threat of antifungal resistance was published during proof corrections.^117^
