# Supplementary material for: Metal Complexes as Antifungals? From a Crowd-Sourced Compound Library to the First In Vivo Experiments
Source: JACS Au. 2022 Sep 23;2(10):2277–94. doi: 10.1021/jacsau.2c00308 (PMC9597602; doi:10.1021/jacsau.2c00308)
Supplement: Supplementary file 1 — au2c00308_si_001.pdf [file au2c00308_si_001.pdf]

# - Supporting Information -

Metal Complexes as Antifungals?

–

From a Crowd-Sourced Compound Library to First *In Vivo* Experiments

Angelo Frei[*a,b], Alysha G. Elliott[a], Alex Kan[c], Hue Dinh[d], Stefan Bräse[e], Alice E. Bruce[f], Mitchell R. Bruce[f], Feng Chen[g], Dhirgam Humaidy[f], Nicole Jung[e], A. Paden King[h], Peter G. Lye[i], Hanna K. Maliszewska[j], Ahmed M. Mansour[k], Dimitris Matiadis[l], María Paz Muñoz[j], Tsung-Yu Pai[c], Shyam Pokhrel[f], Peter J. Sadler[g], Marina Sagnou[l], Michelle Taylor[i], Justin J. Wilson[h], Dean Woods[i], Johannes Zuegg[a], Wieland Meyer[c], Amy K. Cain[d], Matthew A. Cooper[a], Mark A. T. Blaskovich[*a]*

[a] Centre for Superbug Solutions, Institute for Molecular Bioscience, The University of Queensland, St. Lucia, Queensland 4072 (Australia).

[b] Dept. of Chemistry, Biochemistry & Pharmaceutical Sciences, University of Bern, Freiestrasse 3, 3012 Bern, Switzerland

[c] Molecular Mycology Research Laboratory, Centre for Infectious Diseases and Microbiology, Faculty of Medicine and Health, Sydney Medical School, Westmead Clinical School, Sydney Institute for Infectious Diseases, Westmead Hospital-Research and Education Network, Westmead Institute for Medical Research, University of Sydney, Sydney, NSW, Australia

[d] School of Natural Sciences, ARC Centre of Excellence in Synthetic Biology, Macquarie University, Sydney, NSW 2109 (Australia)

[e] Institute of Organic Chemistry, Karlsruhe Institute of Technology, Fritz-Haber-Weg 6, 76131 Karlsruhe (Germany). Institute of Toxicology and Genetics, Karlsruhe Institute of Technology, Hermann-von-Helmholtz-Platz 1, 76344 Eggenstein-Leopoldshafen (Germany).

[f] University of Maine, Department of Chemistry, Orono, ME, USA

[g] Department of Chemistry, University of Warwick, Gibbet Hill Road, Coventry CV4 7AL (UK).

[h] Department of Chemistry and Chemical Biology, Cornell University, Ithaca, NY 14853 (USA).

[i] School of Science and Technology, University of New England, Armidale, NSW 2351, Australia

[j] School of Chemistry, University of East Anglia, Norwich Research Park, Norwich, NR4 7TJ

[k] Chemistry Department, Faculty of Science, Cairo University (Egypt).

[l] Institute of Biosciences & Applications, National Centre for Scientific Research "Demokritos", 15310 Athens, Greece

*angelo.frei@unibe.ch, m.blaskovich@uq.edu.au

Microbial Strain and Other Abbreviations

Table S1. Glossary **of abbreviations** used in this document

G-ve	Gram negative
G+ve	Gram positive
Ab	*Acinetobacter baumannii* ATCC 19606 type strain
Ec	*Escherichia coli* ATCC 25922 FDA control strain
Kp	*Klebsiella pneumoniae* ATCC 700603 ESBL
Pa	*Pseudomonas aeruginosa* ATCC 27853 QC control strain
MRSA	Methicillin resistant *Staphylococcus aureus* ATCC 43300
Ca	*Candida albicans* ATCC 90028 NCCLS11
Cn H99	*Cryptococcus neoformans* H99 ATCC 208821 type strain
HEK	HEK-293 human embryonic kidney cells ATCC CRL-1573
RBC	Human red blood cells
CC50	cytotoxicity concentration 50%
HC10	hemolysis concentration 10% against RBC

Experimental Details

*Synthesis of complexes **Ag1** and **Ag2**.*

A solution of the corresponding pyrazolines[1] (0.16 mmol) in MeOH (1 mL) was added slowly to an aqueous KOH solution (1%, 0.9 mL) at room temperature under stirring. A solution of $AgNO_3$ (27 mg, 0.16 mmol) in H_2O (0.3 mL) was subsequently added in the absence of light. A yellow solid precipitated immediately occurred. The mixture was stirred for 30 more minutes and then, the dark brown solid was filtered using Büchner funnel, washed with ethanol (x2) and water (x2) and dried under vacuum and P_2O_5. The products were protected from light at any time.

*(E)-1-(4-Carboxyphenyl)-5-(3,4-dimethoxyphenyl)-3-(3,4-dimethoxystyryl)-2-pyrazoline silver(I) complex **Ag1***

Dark green solid (45 mg, 48% calculated for 0.08 mmol maximum yield); FT-IR (KBr, cm^{-1}): 1595, 1510, 1375, 1260, 1135, 1025, 770, 515; ^1H NMR (DMSO-d_6) δ: 2.98 (1H, dd, J_{MX} = 4.9 Hz, J_{AM} = 16.9 Hz, CH_AH_M), 3.69 (3H, s, MeO),

3.71 (3H, s, MeO), 3.76 (1H, overlapped, CH_AH$_M$), 3.76 (3H, s, MeO), 3.81 (3H, s, MeO), 5.43 (1H, dd, J_{MX} = 5.1 Hz, J_{AX} = 11.9 Hz, CH_X), 6.68 (1H, d, J = 8.3 Hz, *H28*), 6.73 (1H, d, J = 16.2 Hz, *H16*), 6.87-6.91 (4H, m, *H21, H29*, *H32*), 6.93 (1H, d, J = 8.4 Hz, *H21*), 7.06 (1H, d, J = 8.4 Hz, *H22*), 7.19 (1H, d, J = 16.2 Hz, *H15*), 7.24 (1H, s, *H18*), 7.71 (2H, d, J = 8.6 Hz, *H8*, *H10*); ^{13}C NMR (DMSO-d_6) δ: 41.9 (*C4*), 55.4 (CH_3O-), 55.5 (CH_3O-), 62.2 (*C5*), 109.2 (*C18*), 109.6 (*C32*), 111.6 (*C7*, *C11),* 111.7 (*C29*), 112.2 (*C21*), 117.4 (*C28*), 119.1 (*C15*), 121.7 (*C9*), 120.5 (*C22*), 129.4 (*C17*), 130.8 (*C8*, *C10*), 134.0 (*C27*), 134.5 (*C16*), 145.4 (*C6*), 148.0 (*C30*), 149.0 (*C31*), 149.1 (*C20*), 149.2 (*C19*), 150.3 (*C3*), 170.0 (-*C*OOH).

1-(4-Carboxyphenyl)-3-{(1E,3E)-4-phenylbuta-1,3-dien-1-yl}-5-{(E)-styryl}-2-pyrazoline silver(I) complex ***Ag2***

Dark brown solid (43 mg, 51% calculated for 0.08 mmol maximum yield); FT-IR (KBr, cm^{-1}): 1595, 1535, 1510, 1370, 1320, 1125, 980, 745, 690, 500; ^{1}H NMR (DMSO-d_6) δ: 3.01 (1H, dd, J_{MX} = 5.2 Hz, J_{AM} = 16.8 Hz, CH$_A$$H_M$), 3.51 (1H, dd, J_{AX} = 11.5 Hz, J_{AM} = 16.8 Hz, CH_AH$_M$), 5.19 (1H, ddd, J_{MX} = 5.2 Hz, J_{AX} = 11.5 Hz, J_{XH15b}= 7.6 Hz, CH_X), 6.28 (1H, dd, $J_{H15bH16b}$ = 16.0 Hz, $J_{H15'X}$ = 7.6 Hz, *H-15b*), 6.67 (1H, d, J = 16.0 Hz, *H-16b*), 6.69 (1H, d, J = 15.8 Hz, *H-16*), 6.77 (2H, d overlapped, *H-15a*, *H-16a*), 7.06 (d, 2H, J = 8.7 Hz, *H-7*, *H-11*), 7.13-7.27 (3H, m, *H-15*, *H-20, H-30*), 7.30 (2H, t, J = 7.6 Hz, *H-29*, *H-31*), 7.36 (2H, t, J = 7.6 Hz, *H-19*, *H-21*), 7.43 (2H, d, J = 7.6 Hz, H-28, H-32), 7.52 (2H, d, J = 7.6 Hz, *H-18, H-22*), 7.79 (d, 2H, J = 8.7 Hz, *H-8*, *H-10*); ^{13}C NMR (DMSO-d_6) δ: 38.7 (C-4), 61.2 (C-5), 112.0 (C-7, C-11), 122.7 (C-9), 125.1 (C-15), 126.49 (Ph), 126.52 (C-18, C-22), 127.9 (C-15b), 128.4, 128.6, 128.8, 128.83, 128.93 (Ph), 130.8 (C-8, C-10), 131.2 (C-16b), 134.0, 134.6, 136.0 (C-16, C-15a, C-16a), 136.8 (C-17), 142.3 (C-27), 145.6 (C-6), 150.7 (C-3), 168.4 (-COOH).

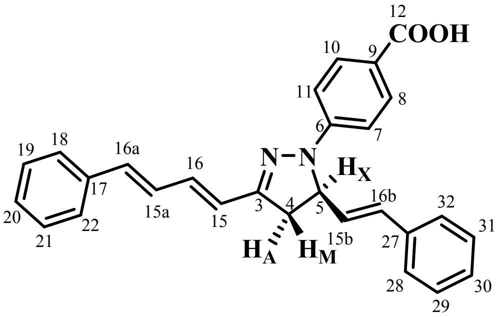

Ligand-Ag2

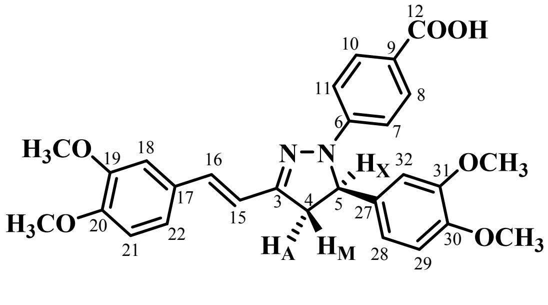

Ligand-Ag1

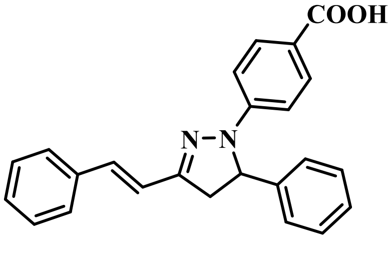

Ligand-Ag3

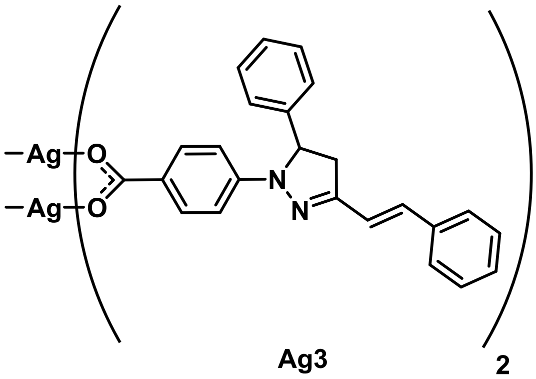

Ag3

Figure S1. Structures of complex **Ag3** and ligands used to prepare the complexes **Ag1**, **Ag2** and **Ag3**. Numbering of compounds **Ligand-S1** and **Ligand-S2** is according to the numbering of published X-ray structure [1] to describe the AMX system and the NMR assignments of the products **Ag1** and **Ag2**. The products are racemic but single molecules are shown for clarity and simplicity purposes.

Table S2. MIC, CC50 and HC10 values for selected **silver** analogues (values given in µg/mL).

	G-ve				G+ve	Fungi				
	Ab	Ec	Kp	Pa	MRSA	Ca	Cn	HEK CC_{50}	RBC HC_{10}	Unit
**Ligand-Ag2#**	>32	>32	>32	>32	>32	>32	>32	n.d.	n.d.	µg/mL
**Ligand-Ag1#**	>32	>32	>32	>32	>32	>32	>32	n.d.	n.d.	µg/mL

**Ligand-Ag3#**	>32	>32	>32	>32	>32	>32	>32	n.d.	n.d.	µg/mL
**Ag3**	>32	>32	>32	>32	>32	16	16	>32	>32	µg/mL
**$AgNO_3$**	≤0.25	≤0.25	≤0.25	≤0.25	2	≤0.25	≤0.25	>32	8.9	µg/mL

#This compound showed no inhibition in the single concentration (32 µg/mL) CO-ADD Primary Screening and was hence not evaluated in a dose-response assay. n.d.: not determined

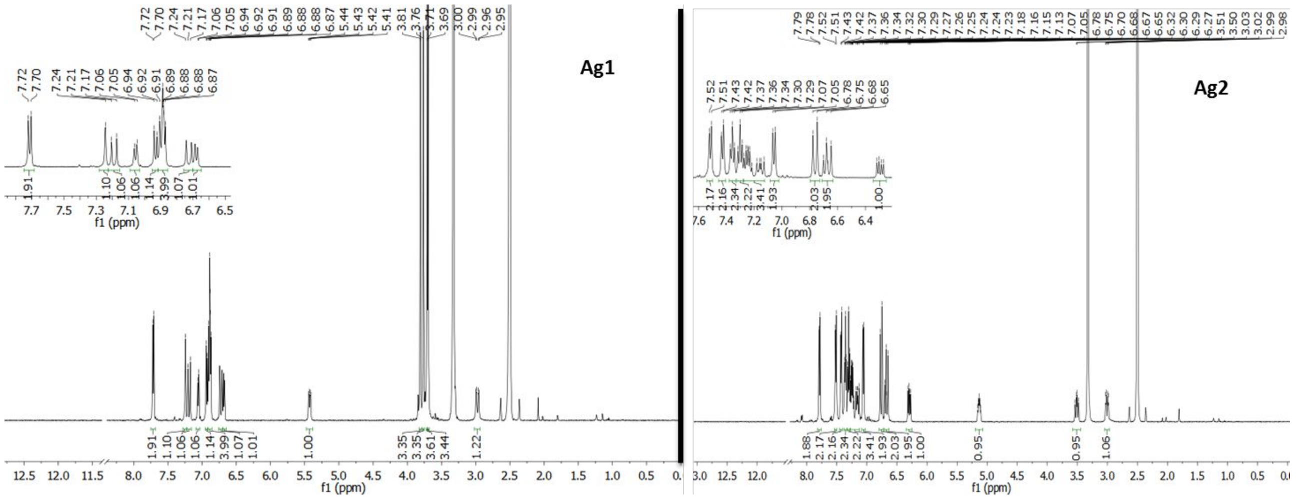

Figure S2. ^{1}H NMR spectra of complexes **Ag1** and **Ag2** in DMSO-d_6.

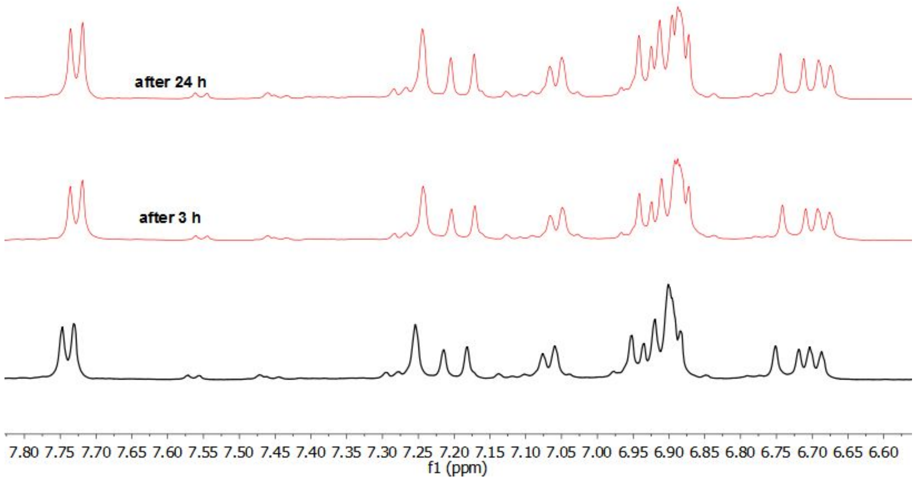

Figure S3. Superimposed ^{1}H NMR spectra of silver(I) complex **Ag1** for stability test – t = 0, 3, 24 h.

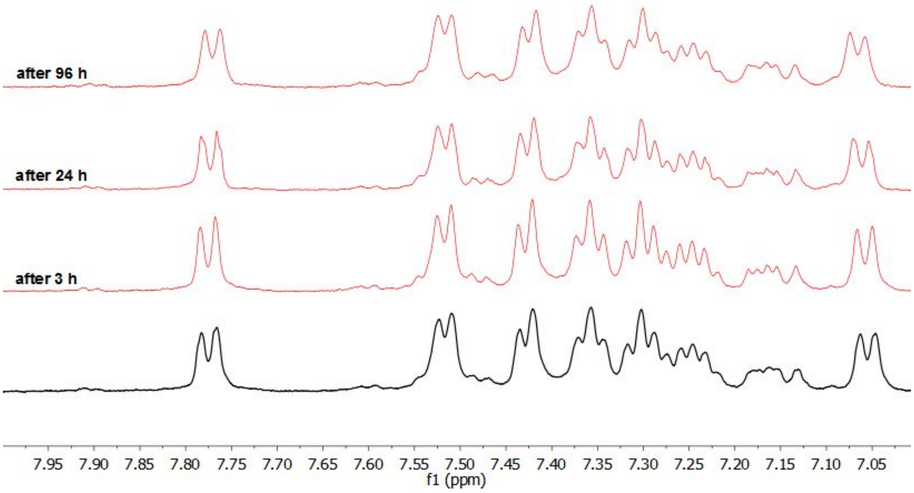

Figure S4. Superimposed ^{1}H NMR spectra of silver(I) complex **Ag2** (red) with ligand (black). For stability – t = 0, 3, 24, 96 h.

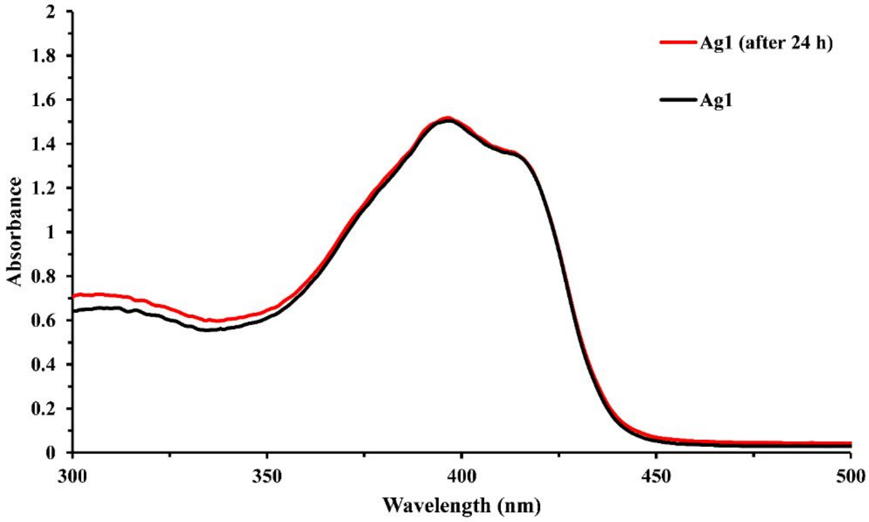

Figure S5. UV-Vis Stability test for compound **Ag1** in 10% DMSO (20 μM) over 24 h.

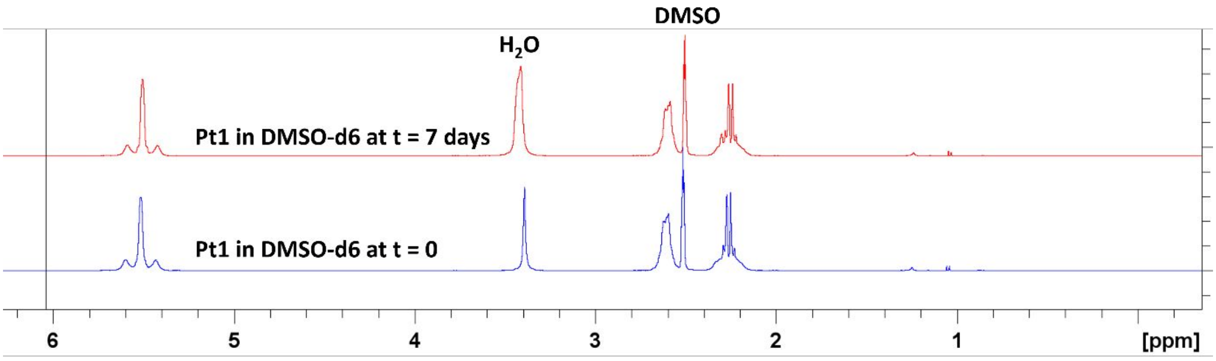

Figure S6. 1H NMR spectrum of **Pt1** in DMSO-d6 after 0 days and after 7 days at room temperature.

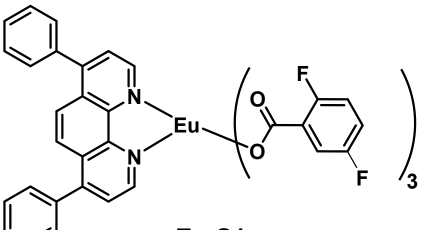

Eu-S1

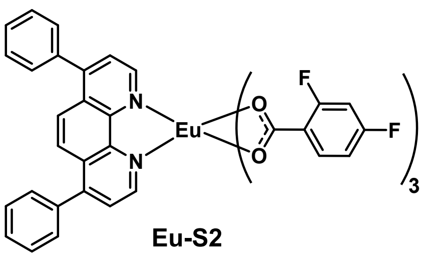

Eu-S2

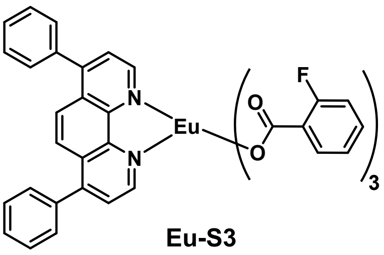

Eu-S3

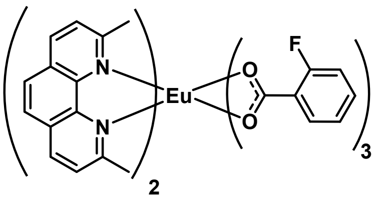

Eu1

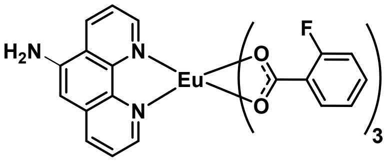

Eu-S4

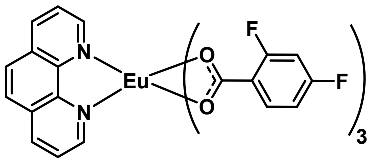

Eu-S5

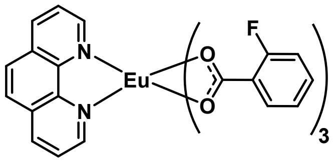

Eu-S6

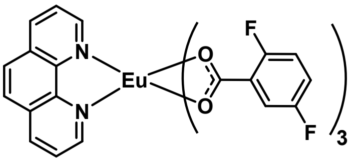

Eu-S7

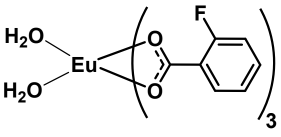

Eu-S8

Figure S7. Structures of selected **europium** analogues.

Table S3. MIC, CC50 and HC10 values for selected **europium** analogues (values given in µg/mL).

	G-ve				G+ve	Fungi				
	Ab	Ec	Kp	Pa	Sa	Ca	Cn	HEK CC_{50}	RBC HC_{10}	Unit
**Eu-S1**	>20	>20	>20	>20	10	>20	5	>20	>20	µM
**Eu-S2**	>20	>20	>20	>20	1.25	10	0.625	15.9	0.7	µM
**Eu-S3**	>20	>20	>20	>20	10	20	5	14.7	>20	µM
**Eu1**	>20	>20	>20	>20	>20	≤0.16	≤0.16	>20	>20	µM
**Eu-S4**	>20	>20	>20	>20	>20	>20	20	3.3	1.2	µM
**Eu-S5**	>20	>20	>20	>20	>20	>20	20	13.6	0.5	µM
**Eu-S6**	>20	>20	>20	>20	>20	20	20	9.9	>20	µM
**Eu-S7#**	n.a.	n.a.	n.a.	n.a.	n.a.	n.a.	n.a.	n.a.	n.a.	µM
**Eu-S8#**	n.a.	n.a.	n.a.	n.a.	n.a.	n.a.	n.a.	n.a.	n.a.	µM

#This compound showed no inhibition in the single concentration (20 µM) CO-ADD Primary Screening and was hence not evaluated in a dose-response assay.

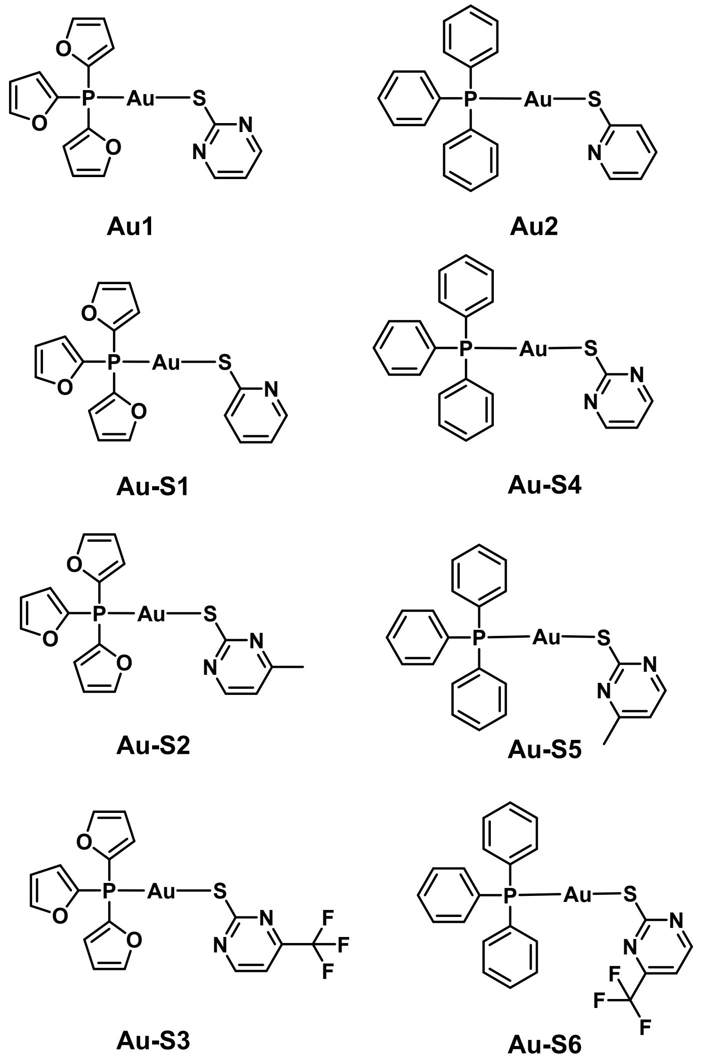

Figure S8. Structures of selected **gold** analogues.

Table S4. MIC, CC50 and HC10 values for selected **gold** analogues (values given in µg/mL).

	G-ve	G+ve	Fungi			

	Ab	Ec	Kp	Pa	Sa	Ca	Cn	HEK CC_{50}	RBC HC_{10}	
**Au1**	n.d.	n.d.	n.d.	>32	≤0.25	≤0.25	≤0.25	>32	>32	µg/mL
**Au-S1**	>32	>32	>32	>32	16	16	16	>32	>32	µg/mL
**Au-S2**	>32	>32	>32	>32	>32	>32	>32	1.38	>32	µg/mL
**Au-S3**	>32	>32	>32	>32	≤0.25	>32	>32	>32	>32	µg/mL
**Au2**	>32	>32	>32	>32	≤0.25	≤0.25	≤0.25	>32	>32	µg/mL
**Au-S4**	>32	>32	>32	>32	>32	>32	>32	>32	>32	µg/mL
**Au-S5**	>32	>32	>32	>32	>32	>32	>32	>32	>32	µg/mL
**Au-S6**	>32	>32	>32	>32	≤0.25	>32	>32	>32	>32	µg/mL

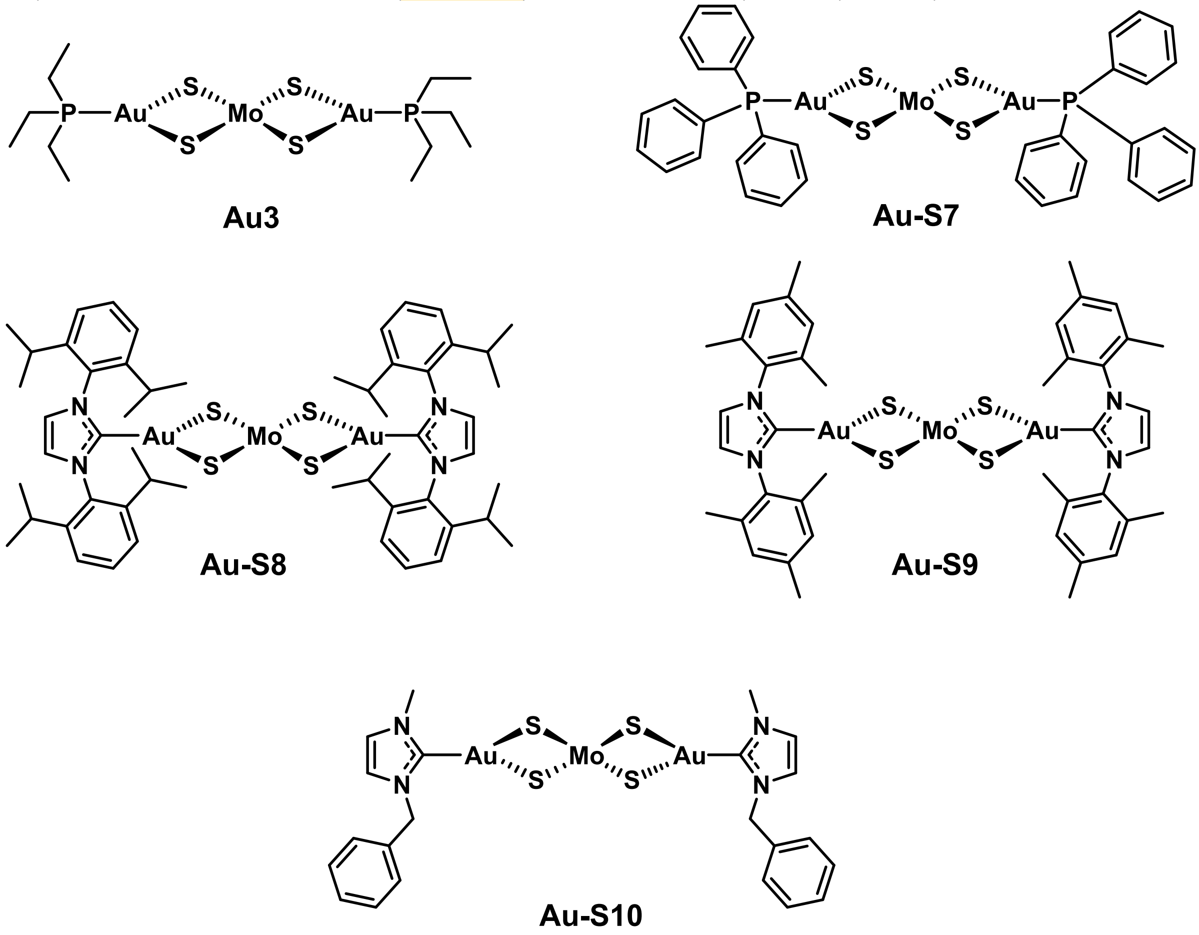

Figure S9. Structures of selected **gold-molybdenum** analogues.

Table S5. MIC, CC50 and HC10 values for selected **gold-molybdenum** analogues (values given in µg/mL).

	G-ve				G+ve	Fungi		HEK	RBC	
	Ab	Ec	Kp	Pa	Sa	Ca	Cn	CC_{50}	HC_{10}	
**Au3**	>32	>32	>32	>32	≤0.25	16	≤0.25	>32	>32	µg/mL
**Au-S7#**	>32	>32	>32	>32	>32	>32	>32	n.d.	n.d.	µg/mL
**Au-S8#**	>32	>32	>32	>32	>32	>32	>32	n.d.	n.d.	µg/mL
**Au-S9#**	>32	>32	>32	>32	>32	>32	>32	n.d.	n.d.	µg/mL
**Au-S10**	>32	>32	>32	>32	>32	≤0.25	>32	>32	>32	µg/mL

#This compound showed no inhibition in the single concentration (32 µg/mL) CO-ADD Primary Screening and was hence not evaluated in a dose-response assay for MIC evaluation. Values shown for these compounds as >32 µg/mL was a single point concentration assay, n=2.

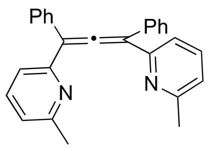

Au-Ligand1

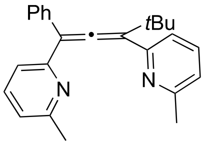

Au-Ligand2

Allene-containing metal complexes:

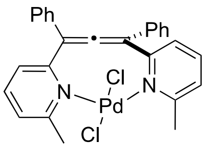

HM_C1

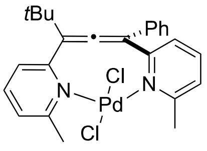

HM_C2

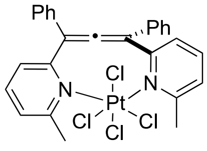

HM_C6

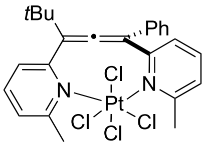

HM_C5

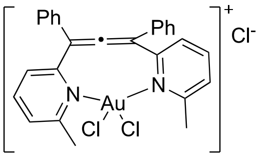

Au-S11

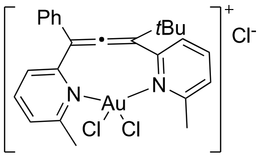

Au-S12

Platinum(**II**) and gold carbene complexes:

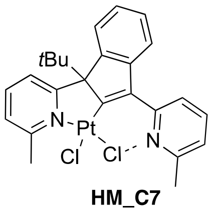

HM_C7

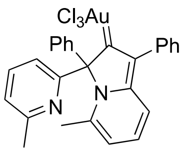

Au-S13

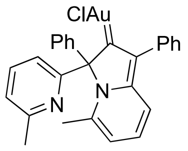

Au-S14

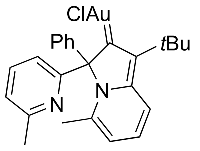

Au-S15

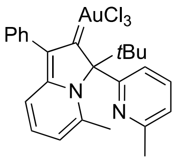

Au4a

Au4

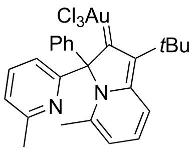

Au4b

Figure S10. Structures of selected **gold/platinum/palladium** analogues.

Table S6. MIC, CC50 and HC10 values for selected **gold/platinum/palladium** analogues (values given in µg/mL).[2]

	G-ve				G+ve	Fungi		HEK	RBC	
	Ab	Ec	Kp	Pa	Sa	Ca	Cn	CC_{50}	HC_{10}	
**Au-Ligand1**	n.a.	n.a.	n.a.	n.a.	n.a.	n.a.	n.a.	n.a.	n.a.	µg/mL
**Au-Ligand1**	n.a.	n.a.	n.a.	n.a.	n.a.	n.a.	n.a.	n.a.	n.a.	µg/mL
**Au-S11**	>32	32	>32	>32	16	4	16	>32	>32	µg/mL
**Au-S12**	>32	>32	>32	>32	16	4	<=0.25	>32	<=0.25	µg/mL
**Au-S13**	32	16	16	>32	<=0.25	2	<=0.25	>32	19.3	µg/mL
**Au-S14**	32	>32	32	>32	<=0.25	<=0.25	<=0.25	0.7	23.7	µg/mL
**Au-S15**	32	>32	32	32	2	<=0.25	<=0.25	>32	>32	µg/mL
**Au4**	32	32	32	>32	0.5	1	<=0.25	>32	>32	µg/mL
**HM_C1**	>32	>32	>32	>32	2	16	1	1.0	>32	µg/mL
**HM_C2**	32	>32	>32	>32	<=0.25	<=0.25	<=0.25	1.1	>32	µg/mL
**HM_C5**	>32	>32	>32	>32	>32	32	>32	>32	>32	µg/mL

**HM_C6**	n.a.	n.a.	n.a.	n.a.	n.a.	n.a.	n.a.	n.a.	n.a.	μg/mL
**HM_C7**	>32	>32	>32	>32	2	4	2	14.5	>32	μg/mL

Table S7. Cytotoxicity (CC_{50}) and haemolysis (HC_{10}) ranges obtained for highly active compounds upon retesting. Therapeutic Index (**TI**) with respect to cytotoxicity (**TI_CC50)** and haemoysis (**TI_HC10**) is given against the best MIC measured. The **Minimum TI** is the lowest TI obtained in either TI_CC50 or TI_HC10. Highlighted compounds were advanced to *in vivo* toxicity screening in *G. mellonella* based on TI and compound availability.

	**HEK293 CC50**	**RBC HC10**	**TI_CC50**	**TI_HC10**	**Minimum TI**
**Co1**	>100	>100	256	256	256
**Ni1**	>200	>200	80	80	80
**Rh1**	>200	0.4-3	513	1-9	1
**Pd1**	>100	10	128	13	13
**Pd2**	38-53	0.2-3	1563 - 2192	10-130	10
**Pd3**	89-123	17-56	14,733 - 20,433	2900-9350	2900
**Ag1**	3-4	13-154	417 - 683	2117-25,600	2117
**Ag2**	6-7	>200	933 -1117	33,333	933
**Eu1**	>200	>200	33,333	33,333	33,333
**Ir1**	73-96	23-200	3029 - 3992	8333	3029
**Ir2**	62-65	2-76	2600 - 2717	3154	2600
**Ir3**	54-76	3-41	4450 - 6300	3383	3383
**Pt1**	>100	>100	64	64	64
**Pt2**	>100	>100	128	128	128
**Pt3**	>100	>100	8	8	8
**Pt4**	>100	>100	128	128	128
**Pt5**	16-130	>200	2667 - 21,667	33,333	2667
**Au1**	0.1-0.6	90-200	5 - 25	3746-8333	5
**Au2**	0.8-200	>200	35 - 8333	8333	35
**Au3**	139-170	200	44 - 54	64	44
**Au4**	200	200	33,333	33,333	33,333

Table S8. Molecular Formula and Molecular Weight for highly active compounds highlighted in the paper.

ID	Molecular Formula	MW
**Co1**	C12H18CoN11O3S2	487.4037
**Ni1**	C44H34B2F8N6Ni	705.4914
**Rh1**	C20H30Cl4Rh2	618.0710
**Pd1**	C8H12Cl2Pd	285.5040
**Pd2**	C26H24N2O6Pd2S2	737.4460
**Pd3**	C23H21Cl3N5Pd1.5	633.4365
**Ag1**	C64H62Ag2N4O12	1190.80
**Ag2**	C56H46Ag2N4O4	1054.74
**Eu1**	C49H36EuF3N4O6	985.8082
**Ir1**	C38H42BrClIrN5O2S	940.4180
**Ir2**	C36H36ClIrN6O4S	876.4490
**Ir3**	C42H44ClIrN6O2S	924.5810
**Pt1**	C8H12Cl2Pt	374.1680
**Pt2**	C8H12I2Pt	557.0769
**Pt3**	C36H36ClIrN6O4S	876.4490
**Pt4**	C12H19ClOPt	409.8170
**Pt5**	C23H21Cl3N5Pt1.5-	766.4325
**Au1**	C16H12AuN2O3PS	540.2833
**Au2**	C23H19AuNPS	569.4123
**Au3**	C12H30Au2MoP2S4	854.4437
**Au4**	C25H26AuCl3N2	657.8136

Table S9. Extended panel MICs (*50% inhibition, MIC score 2, μM)

MIC*	*Candida albicans*	*Candida auris*	*Candida auris*	*Candida glabrata*	*Candida tropicalis*	*Cryptococcus deuterogattii*	*Cryptococcus deuterogattii*	*Cryptococcus neoformans*
	ATCC 90028	CBS10913	CBS12373	ATCC 90030	ATCC 750	CBS7750	ATCC 32609	ATCC 208821
	NCCLS 11	JCM 15448;DSM 21092; CBS 10913	KCTC 17810	NCCLS 84	QC strain for susceptibility testing	VGII; Serotype B	VGII	H99; VN1
**ID**	**MIC50 [µM] ***							
**Co1**	[redacted]							
**Ni1**	>160	≥160	2.5 - 20	≥160	160	5 - 10	5 - 10	2.5 - 5
**Rh1**	0.391 - 1.56	0.781- 1.56	0.391 - 0.781	0.391 - 6.25	0.391 - 0.781	0.391	0.78 - 1.56	0.195 - 0.391
**Pd1**	[redacted]							
**Pd2**	0.049 - 0.195	0.024 - 0.098	0.024 - 0.049	0.012 - 0.195	0.195 - 0.391	≤0.006	0.024 - 0.098	≤0.006 - 0.024
**Pd3**	0.012 - 0.024	≤0.006	≤0.006	0.012 - 0.024	≤0.006 - 0.024	0.012 - 0.024	0.012 - 0.024	0.012 - 0.049
**Ag1**	≤0.006	≤0.006	≤0.006	≤0.006	≤0.006	≤0.006	≤0.006 - 0.024	≤0.006
**Ag2**	≤0.006 - 0.098	≤0.006 - 0.049	≤0.006 - 0.098	0.006 - 0.195	≤0.006 - 0.049	≤0.006 - 0.049	≤0.006 - 0.098	≤0.006 - 0.098
**Eu1**	≤0.006 - 0.012	≤0.006	≤0.006	0.012 - 0.049	≤0.006 - 0.049	0.024 - 0.098	0.024	≤0.006
**Ir1**	0.049 - 0.098	0.098	0.049	0.098 - 0.195	0.098 - 0.195	0.024	0.098 - 0.195	0.024 - 0.049
**Ir2**	0.049 - 0.098	0.049 - 0.098	0.024	0.098 - 0.195	0.098 - 0.195	0.012 - 0.024	0.098 - 0.195	0.024 - 0.098
**Ir3**	0.049	0.098	0.024 - 0.049	0.098	0.098	0.024	0.049 - 0.098	0.024
**Pt1**	[redacted]							
**Pt2**	[redacted]							
**Pt3**	[redacted]							
**Pt4**	[redacted]							
**Pt5**	≤0.006 - 0.049	≤0.006 - 0.024	≤0.006 - 0.024	≤0.006 - 0.024	≤0.006 - 0.024	≤0.006 - 0.098	≤0.006 - 0.049	≤0.006 - 0.049
**Au1**	0.049 - 0.391	0.098 - 0.195	0.098 - 0.391	0.098 - 0.391	0.049 - 0.391	0.024 - 0.098	0.024 - 0.049	0.098 - 0.195
**Au2**	0.098 - >200	0.098 - 1.56	0.098 - 0.195	>200	≥200	0.049 - 1.56	0.049 - 0.195	0.024 - 0.195
**Au3**	3.13 - 200	3.13 - 6.25	3.13 - 200		6.25 - 100	3.12 - 100	3.12 - 6.25	3.12 - 6.25
**Au4***	0.195 - 0.391	0.195 - 0.391	≤0.006 - 0.049	0.781 - 6.25	1.56 - 6.25	0.098 - 0.195	0.024 - 0.049	0.098 - 0.195

*Two new samples of Au4 (1:2 and 1:0.7 (Au4a:Au4b)) were received for further testing. Both mixtures gave the same MIC values across all assays. NA: MIC value not available due to wide replicate variation.

Table S10. Comparator MICs of antifungals, μg/mL

	Candida albicans	*Candida auris*	*Candida auris*	*Candida glabrata*	*Candida tropicalis*
	ATCC 90028	CBS10913	CBS12373	ATCC 90030	ATCC 750
**(*) MIC = ≥80% inhibition**	NCCLS 11	JCM 15448	KCTC 17810	NCCLS 84	QC strain for susceptibility testing
**ID**	**MIC* [μg/mL]**				
Itraconazole	0.062 - 0.25	0.063	2	32	2
Amphotericin B	0.125 - 1	16	>128	≥64	2 - 4
Micafungin	≤0.0005	≤0.001	2	4 - 32	0.008 - 0.063
5-Fluorocytosine	≤0.001	≤0.002	≤0.002	>32	0.001 - 0.002
Posaconazole	0.001 - 0.031	0.031 - 0.063	2	2	0.125
Ketoconazole	0.002 - 0.004	0.004 - 0.016	≤0.004	NT	NT
Fluconazole	0.004	≤0.004	≤0.004	NT	NT
Voriconazole	0.008	0.004 - 0.008	0.156	NT	NT
Caspofungin	0.391 - 1.56	6.25	12.5	6.25 - 12.5	1.56
Anidulafungin	0.031 - 0.125	0.031 - 0.125	0.25	0.008	0.004 - 0.008

	Cryptococcus deuterogattii	*Cryptococcus deuterogattii*	*Cryptococcus neoformans*
	CBS7750	ATCC 32609	ATCC 208821
**(*) MIC = ≥80% inhibition**	VGII; Serotype B	VGII	H99; VN1

ID	MIC* [µg/mL]		
Itraconazole	2 - 4	2 - 8	1 - 2
Amphotericin B	1 - 8	4 - 16	2 - 8
Micafungin	0.06 - 0.125	0.016 - 0.125	0.015 - 0.063
5-Fluorocytosine	0.06 - 0.25	0.002 - 0.25	0.001 - 0.016
Posaconazole	0.06 - 0.125	0.015 - 0.25	0.016 - 0.125
Ketoconazole	NT	NT	>64
Fluconazole	NT	NT	2 - 4
Voriconazole	NT	NT	≥64
Caspofungin	0.06 - 0.1	0.1 - 0.63	0.098 - 1.56
Anidulafungin	0.06 - 0.25	0.003 - 0.25	0.016 - 0.125

Table S11. Comparator MICs of antifungals, μM

	Candida albicans	*Candida auris*	*Candida auris*	*Candida glabrata*	*Candida tropicalis*
	ATCC 90028	CBS10913	CBS12373	ATCC 90030	ATCC 750
**(*) MIC = ≥80% inhibition**	NCCLS 11	JCM 15448	KCTC 17810	NCCLS 84	QC strain for susceptibility testing
**ID**	**MIC* [µM]**				
Itraconazole	0.089 - 0.354	0.089	2.83	45	2.83
Amphotericin B	0.192 - 1.53	24.5	≥196	≥98.2	3.01 - 6.13
Micafungin	≤0.0007	≤0.0014	2.23	4.45 - 35.7	0.009 - 0.070
5-Fluorocytosine	≤0.012	≤0.021	≤0.021	>351	0.012 - 0.021
Posaconazole	0.002 - 0.063	0.063- 0.126	4.04	4.04	0.253
Ketoconazole	0.005 - 0.010	0.010 - 0.042	≤0.01	NT	NT
Fluconazole	0.018	≤0.018	≤0.018	NT	NT
Voriconazole	0.032	0.016 - 0.032	0.447	NT	NT
Caspofungin	0.357 - 1.43	5.72	11.4	5.72 - 11.4	1.43
Anidulafungin	0.027 - 0.110	0.027 - 0.110	0.219	0.007	0.003 - 0.007

	Cryptococcus deuterogattii	*Cryptococcus deuterogattii*	*Cryptococcus neoformans*
	CBS7750	ATCC 32609	ATCC 208821
**(*) MIC = ≥80% inhibition**	VGII; Serotype B	VGII	H99; VN1
**ID**	**MIC* [µM]**		
Itraconazole	2.83 - 5.67	2.83 - 11.3	1.42 - 2.83
Amphotericin B	1.53 - 12.3	6.13 - 24.5	3.01 - 12.3

Micafungin	0.070 - 0.139	0.017 - 0.139	0.017 - 0.070
5-Fluorocytosine	0.686 - 2.75	0.021 - 2.75	0.0107 - 0.172
Posaconazole	0.126 - 0.253	0.032 - 0.506	0.032 - 0.253
Ketoconazole	NT	NT	>170
Fluconazole	NT	NT	9.25 - 18.5
Voriconazole	NT	NT	≥260
Caspofungin	0.029 - 0.089	0.089 - 0.057	0.089 - 1.43
Anidulafungin	0.055 - 0.219	0.003 - 0.219	0.014 - 0.110

Table S12. Antifungal MW (g/mol) used for concentration conversion.

ID	Abbreviation	Class	MW
Itraconazole	ITC/ITR	azole	705.64
Amphotericin B	AMB	polyene	924.079
Micafungin	MFG/MICA	echinocandins	1270.28
5-Fluorocytosine	5FC	pyrimidine	129.093
Posaconazole	POS	azole	700.8
Ketoconazole	KCZ/KTC	azole	531.43
Fluconazole	FCZ/FLC	azole	306.271
Voriconazole	VRC/VOR	azole	349.311
Caspofungin	CAS	echinocandins	1093.3
Anidulafungin	AFG/ANI	echinocandins	1140.2

Table S13. Characterization Data reference for the highly active compounds highlighted in the manuscript.

Compound	Data	Reference
**Co1**	Published, Complex **1**	https://doi.org/10.1002/chem.202003545
**Ni1**	Added below	unpublished
**Rh1**	Published (common synthon)	e.g. https://doi.org/10.1002/ejic.201700199
**Pd1**	Published, **Pd1**	https://doi.org/10.1002/cmdc.202100157
**Pd2**	Added below	https://doi.org/10.14272/reaction/SA-FUHFF-UHFFFADPSC-HWRAKXKBWA-UHFFFADPSC-NUHFF-LUHFF-NUHFF-ZZZ
**Pd3**	published Complex **1**	https://doi.org/10.1039/D1RA06559A
**Ag1**	Added above	unpublished
**Ag2**	Added above	unpublished
**Eu1**	Added below	https://doi.org/10.14272/reaction/SA-FUHFF-UHFFFADPSC-XSDMQMVIDC-UHFFFADPSC-NUHFF-KUHFF-NUHFF-ZZZ
**Ir1**	Published, **11**	https://pubs.acs.org/doi/10.1021/acs.jmedchem.8b00906
**Ir2**	Published, **13**	https://pubs.acs.org/doi/10.1021/acs.jmedchem.8b00906
**Ir3**	Published, **14**	https://pubs.acs.org/doi/10.1021/acs.jmedchem.8b00906
**Pt1**	Published, **Pt1**	https://doi.org/10.1002/cmdc.202100157

**Pt2**	Published, **Pt2**	https://doi.org/10.1002/cmdc.202100157
**Pt3**	Published **Pt9**	https://doi.org/10.1002/cmdc.202100157
**Pt4**	Published **Pt 8**	https://doi.org/10.1002/cmdc.202100157
**Pt5**	Published, 2	https://doi.org/10.1039/D1RA06559A
**Au1**	Added below	unpublished
**Au2**	Added below	unpublished
**Au3**	Added below	unpublished
**Au4***	Published, **3b**	https://pubs.rsc.org/en/content/articlelanding/2020/dt/d0dt00665c

Characterization Data for unpublished compounds

Ni1. ^{1}H NMR (500 MHz, DMSO-d_6): δ 5.46 (d, J = 15.75 Hz, 4H, CH_2), 5.46 (d, J = 15.75 Hz, 4H, CH2), 6.01 (d, J = 15.65 Hz, 4H, CH_2), 7.35-7.37 (m, 8H, Ar-H), 7.50-7.52 (m, 8H, Ar-H), 7.98 (d, J = 7.75 Hz, 4H, Pyr-*m*-H), 8.19 (d, J = 7.75 Hz, 4H, Pyr-*m*-H) ppm.

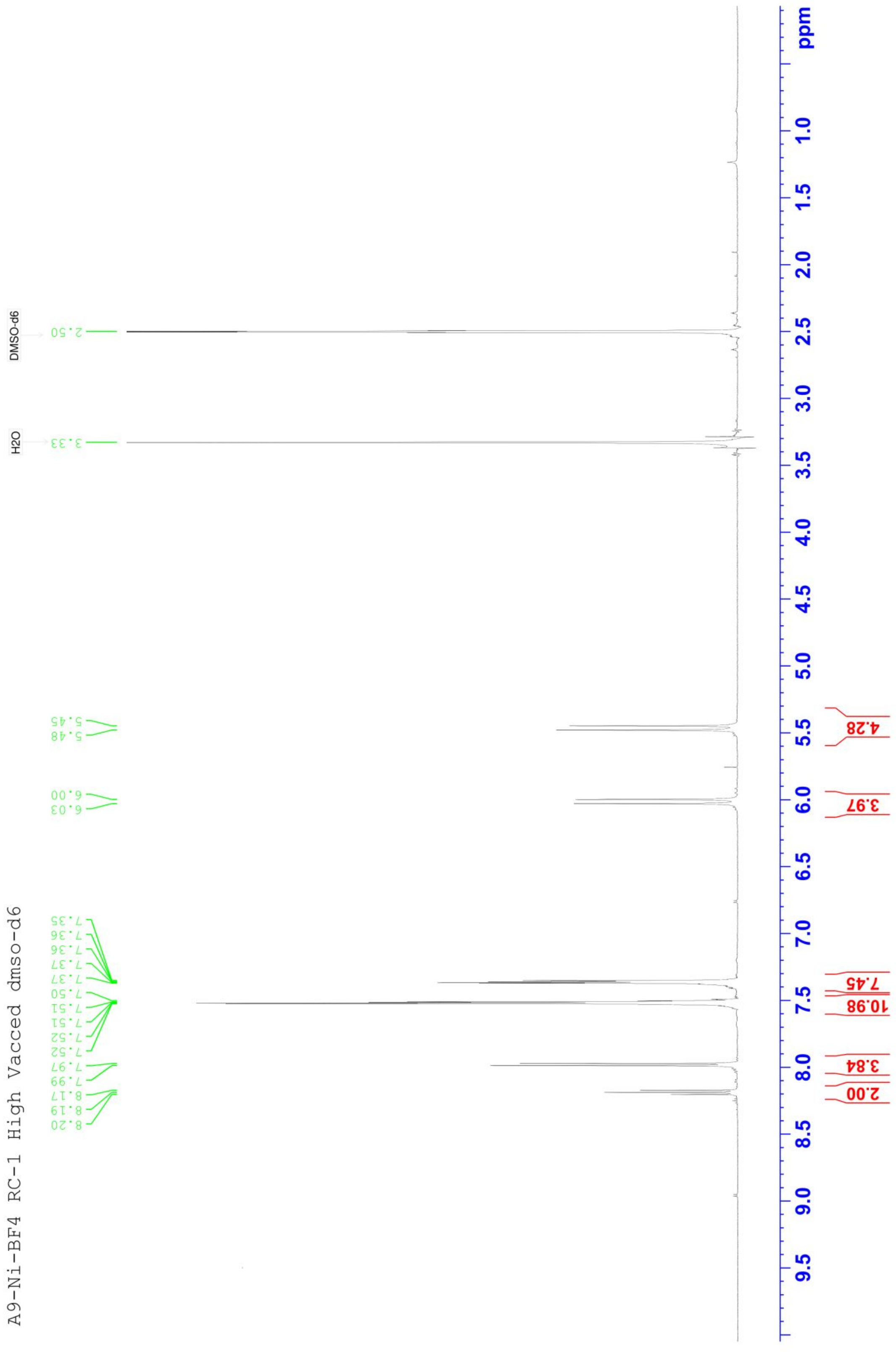

Figure S11. 1H NMR spectrum of **Ni1** in DMSO-d6.

Pd2.

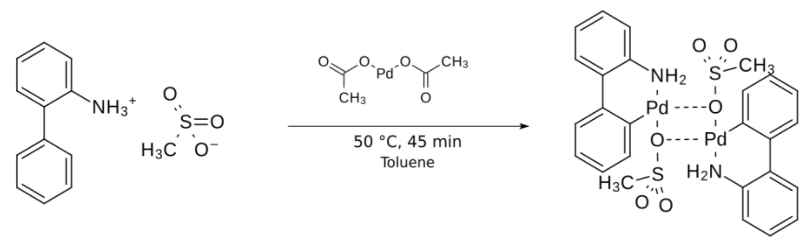

Formula: $C_{26}H_{26}N_2O_6Pd_2S_2$;

Smiles: Nc1ccccc1c1ccccc1[Pd]OS(=O)(=O)C.Nc1ccccc1c1ccccc1[Pd]OS(=O)(=O)C

InChIKey: HWRAKXKBWAQMFD-UHFFFAOYSA-L

A 20 mL Schlenk flask was charged with methanesulfonic acid;2-phenylaniline (500 mg, 1.88 mmol, 1.00 equiv) and palladium(II) acetate (423 mg, 1.88 mmol, 1.00 equiv). Dry toluene (8.00 mL) was added and the mixture was stirred at 50 °C for 45 min until it became milky and brown in appearance. After cooling to 21 °C the suspension was filtered, washed with toluene (1.5 mL) and diethyl ether (3 x 1.5 mL) and dried under vacuum for 24 h. The isolated product Pd2 ($C_{26}H_{26}N_2O_6Pd_2S_2$)) was obtained as a grey solid in 44% yield (609 mg, 824 µmol).

^{1}H NMR (400 MHz, Methanol-d4 [3.31 ppm], ppm) δ = 7.62 (dd, *J* = 1.3 Hz, *J* = 7.3 Hz, 2H), 7.46 (d, *J* = 7.2 Hz, 2H), 7.31–7.21 (m, 6H), 7.16–7.11 (m, 2H), 7.07–7.04 (m, 4H), 2.72 (s, 6H). Missing two H from 2 x NH_2 (4H); ^{13}C NMR (100 MHz, Methanol-d4 [49.1 ppm], ppm) δ = 140.1 (2C), 139.7 (2C), 138.4 (4C), 137.6 (2C), 134.8 (2C), 129.0 (2C), 128.8 (2C), 127.9 (2C), 127.2 (2C), 125.7 (2C), 120.9 (2C), 39.7 (2C). Impurities at 130.0, 129.4, 126.4 ppm; IR (ATR, ṽ) = 3259 (w), 3210 (w), 3054 (w), 3020 (w), 1612 (vw), 1571 (w), 1497 (w), 1465 (vw), 1439 (w), 1425 (w), 1332 (vw), 1320 (vw), 1234 (vs), 1181 (w), 1167 (w), 1160 (w), 1126 (vs), 1106 (vs), 1061 (w), 1048 (w), 1024 (vs), 1001 (w), 983 (w), 965 (w), 941 (vw), 932 (vw), 868 (vw), 827 (vw), 775 (m), 759 (s), 739 (vs), 728 (m), 713 (w), 663 (vw), 615 (vw), 591 (m), 567 (w), 551 (vs), 511 (vs), 470 (s), 452 (w), 405 (w), 387 (w) cm^{-1}.

Additional information on the chemical synthesis is available via Chemotion repository:

https://doi.org/10.14272/reaction/SA-FUHFF-UHFFFADPSC-HWRAKXKBWA-UHFFFADPSC-NUHFF-LUHFF-NUHFF-ZZZ

Additional information on the analysis of the target compound is available via Chemotion repository:

https://doi.org/10.14272/HWRAKXKBWAQMFD-UHFFFAOYSA-L.1

Eu1.

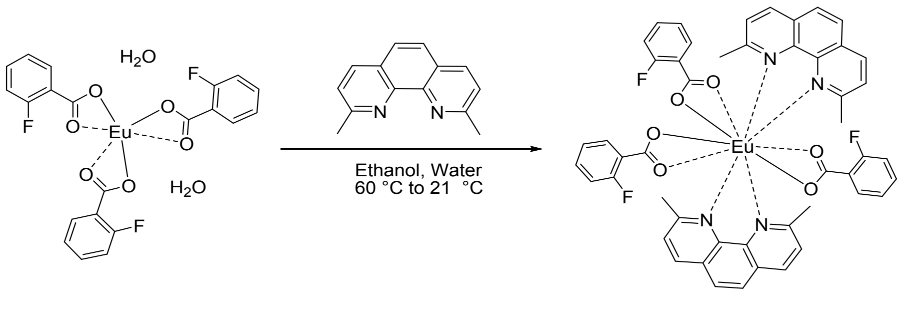

EU1 (75 %)

Formula: $C_{49}H_{36}EuF_3N_4O_6$; Smiles: O=C(c1ccccc1F)O[Eu](OC(=O)c1ccccc1F)OC(=O)c1ccccc1F.Cc1ccc2c(n1)c1nc(C)ccc1cc2.Cc1ccc2c(n1)c1nc(C)ccc1cc2

InChIKey: XSDMQMVIDCRHRR-UHFFFAOYSA-K

A solution of 2,9-dimethyl-1,10-phenanthroline (86.7 mg, 416 µmol, 2.00 equiv) in ethanol (12.0 mL) was slowly added to a solution of tris-europium-2-fluorobenzoate dihydrate (126 mg, 208 µmol, 1.00 equiv) in ethanol (12.0 mL) and water (4.0 mL) and the reaction mixture was heated to 60 °C for 1 hour and then allowed to cool to 21 °C. The precipitate was gained by filtration, washed with cold ethanol and dried in vacuo. The isolated product EU1 (C49H36EuF3N4O6) was obtained as a light brown solid in 75% yield (154 mg, 156 µmol).

^{1}H NMR (400 MHz, DMSO-d6 [2.50 ppm], ppm) δ = 8.33 (d, *J* = 8.2 Hz, 4H), 7.86 (s, 4H), 7.61 (d, *J* = 8.2 Hz, 4H), 7.18 (bs, 4H), 6.74 (bs, 8H), 2.78 (s, 12H); ^{13}C NMR (100 MHz, DMSO-d6 [39.5 ppm], ppm) δ = 160.6 (d, J = 254 Hz, 3C), 158.3 (4C), 144.7 (4C), 136.4 (4C), 132.4 (d, J = 6.9 Hz), 131.6 (4C), 126.6 (4C), 125.5 (4C), 123.4 (4C), 123.1 (d, J = 2.3 Hz, 3C), 114.6 (d, J = 20.8 Hz, 3C), 25.0 (4C). Missing signals due to line broadening (7 C); ^{19}F NMR (376 MHz, ppm) δ = –112.08; IR (ATR, ṽ) = 2918 (w), 1706 (w), 1609 (vs), 1594 (vs), 1545 (vs), 1496 (s), 1486 (s), 1449 (s), 1400 (vs), 1298 (m), 1261 (m), 1220 (vs), 1159 (m), 1145 (m), 1094 (s), 1028 (s), 958 (m), 863 (vs), 809 (s), 759 (vs), 734 (s), 696 (s), 681 (m), 657 (vs), 642 (s), 571 (m), 547 (s), 523 (m), 499 (m), 450 (m), 418 (m), 411 (m), 391 (m), 380 (m) cm^{-1}.

Additional information on the chemical synthesis is available via Chemotion repository:

https://doi.org/10.14272/reaction/SA-FUHFF-UHFFFADPSC-XSDMQMVIDC-UHFFFADPSC-NUHFF-KUHFF-NUHFF-ZZZ

Additional information on the analysis of the target compound is available via Chemotion repository:

https://doi.org/10.14272/XSDMQMVIDCRHRR-UHFFFAOYSA-K.1

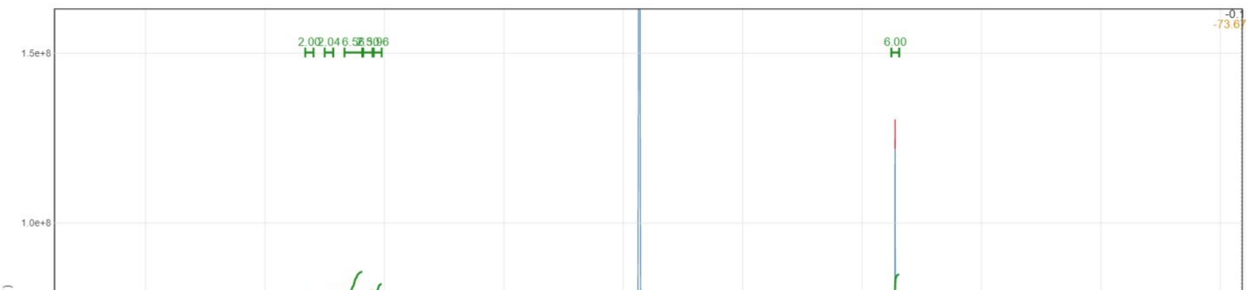

Figure S12. 1H NMR spectrum of **Pd2** in CD_3OD.

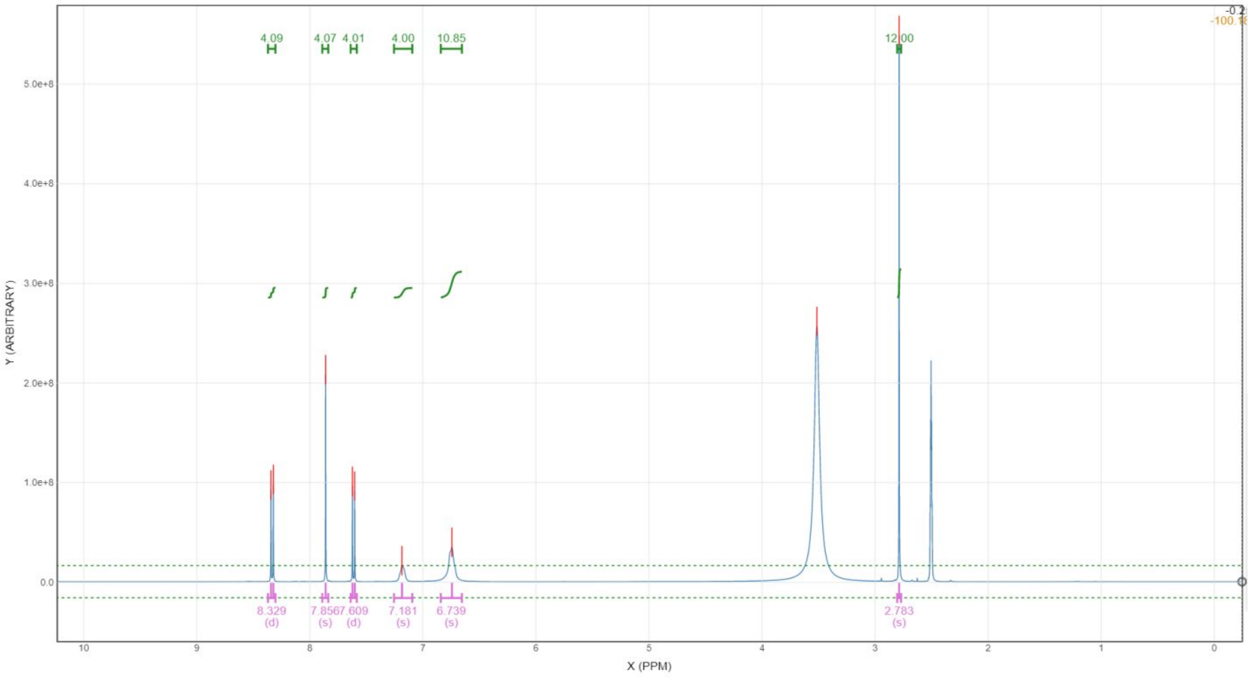

Figure S13. 1H NMR spectrum of **Eu1** in DMSO-d6.

Au1.

MS: Mass-to-charge ratio (*m/z*) values for observed ions with relative abundance (RA) > 10% in ESI mass spectrum: 541.0045 $[M+H]^+$ (RA 100%)

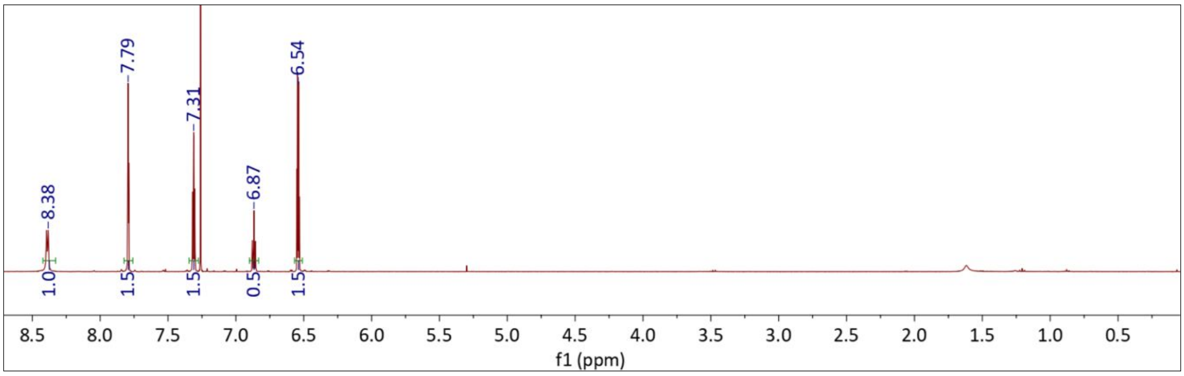

Figure S14. 1H NMR spectrum of **Au1** in $CDCl_3$.

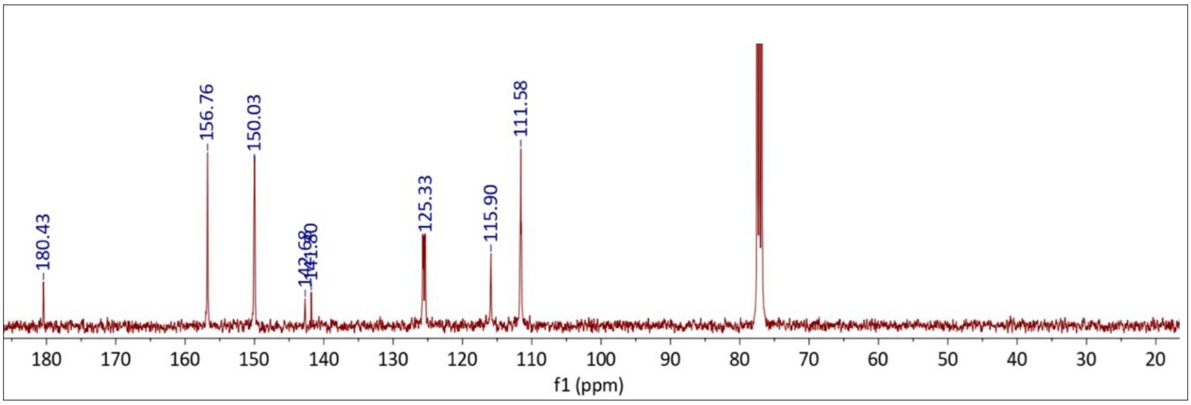

Figure S15. 13C NMR spectrum of **Au1** in $CDCl_3$.

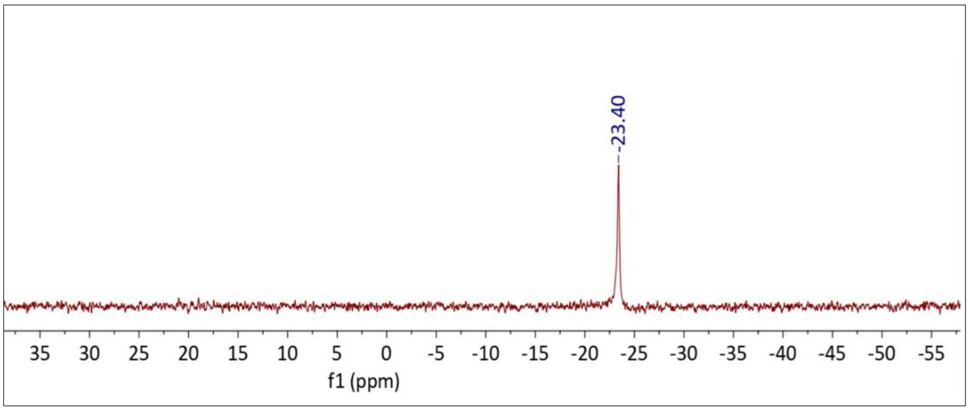

Figure S16. 31P NMR spectrum of **Au1** in $CDCl_3$

Au2.

MS for $C_{23}H_{19}NPSAu$: Mass-to-charge ratio (*m/z*) values for observed ions with relative abundance (RA) > 10% in ESI mass spectrum: 570.0714 $[M+H]^+$ (RA 100%)

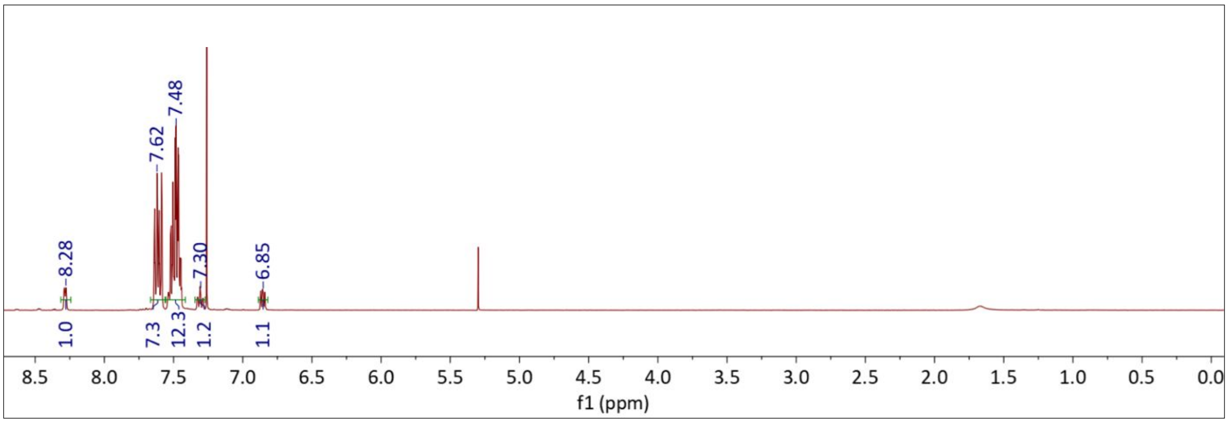

Figure S17. 1H NMR spectrum of **Au2** in $CDCl_3$.

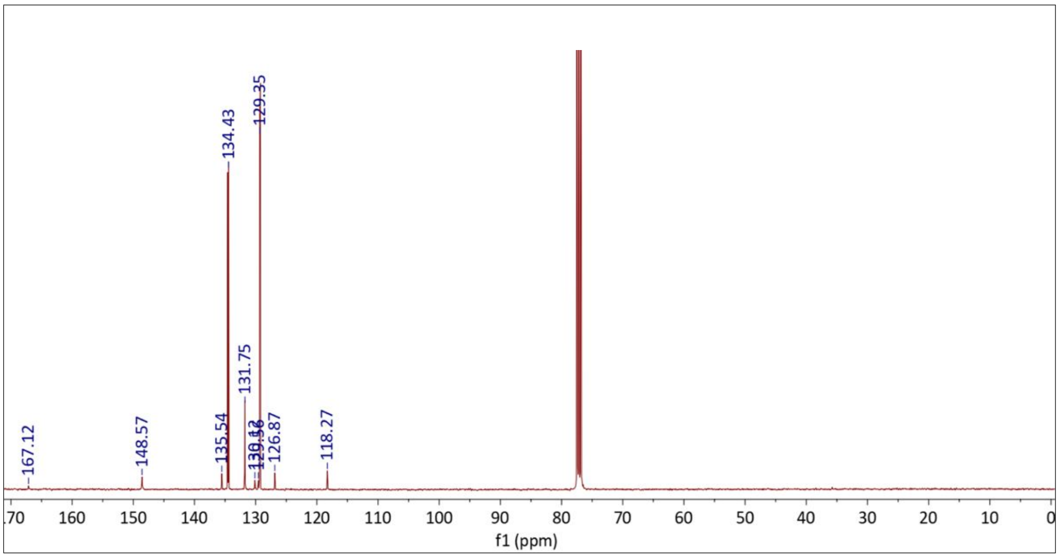

Figure S18. 13C NMR spectrum of **Au2** in $CDCl_3$.

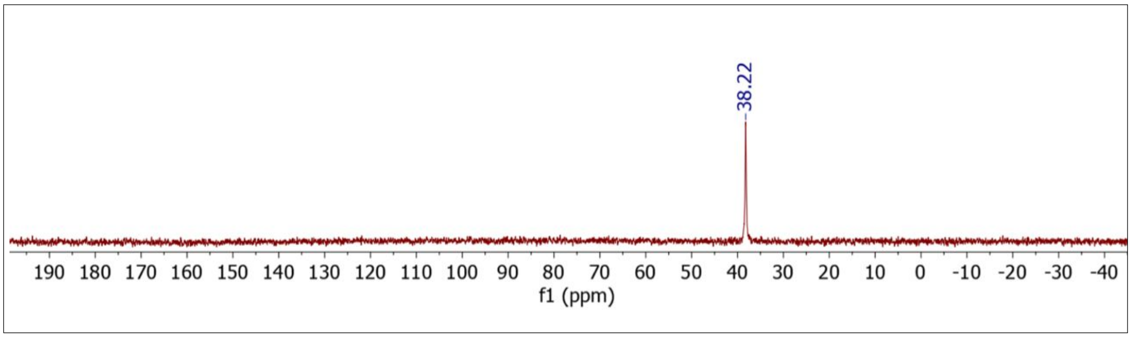

Figure S19. 31P NMR spectrum of **Au2** in $CDCl_3$.

Au3.

EA for $C_{12}H_{30}S_4P_2Au_2Mo$

Experimental % (calculated %)

C: 16.96 (16.87); H: 3.57 (3.54)

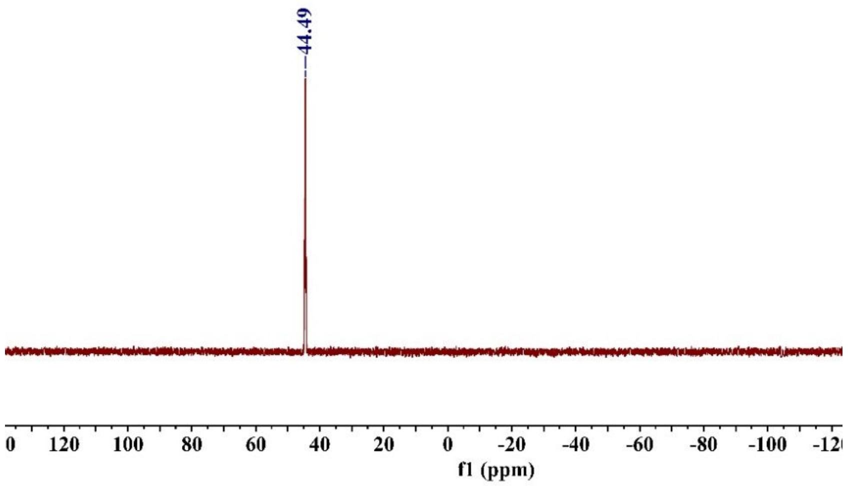

Figure S20. 31P NMR spectrum of **Au3** in DMSO-d6.

References

1. Matiadis, D.; Nowak, K. E.; Alexandratou, E.; Hatzidimitriou, A.; Sagnou, M.; Papadakis, R., Synthesis and (Fluoro)Solvatochromism of Two 3-Styryl-2-Pyrazoline Derivatives Bearing Benzoic Acid Moiety: A Spectral, Crystallographic and Computational Study. *J. Mol. Liq.* **2021,** *331*, 115737.
2. Maliszewska, H. K.; Arnau del Valle, C.; Xia, Y.; Marín, M. J.; Waller, Z. A. E.; Muñoz, M. P., Precious Metal Complexes of Bis(Pyridyl)Allenes: Synthesis and Catalytic and Medicinal Applications. *Dalton Trans.* **2021,** *50* (45), 16739-16750.
